# Continuous Flow Photocatalysis for Sustainable Chemical Conversions

**DOI:** 10.1021/jacsau.5c01612

**Published:** 2026-03-09

**Authors:** Sitong Feng, Wanjun Shi, Tingbin Lim, Emma Richards, Ren Su

**Affiliations:** † Soochow Institute for Energy and Materials InnovationS (SIEMIS), 12582 Soochow University, Suzhou 215006, China; ‡ Joint School of National University of Singapore and Tianjin University, 12605 International Campus of Tianjin University, Bin-hai New City, Fuzhou 350207, China; § School of Chemistry, Cardiff University, Park Place, Cardiff CF10 3AT, U.K.

**Keywords:** continuous flow chemistry, heterogeneous photocatalysis, homogeneous photocatalysis, reactor design, sustainable synthetic chemistry, scale-up

## Abstract

Photocatalytic chemical conversion shows a bright future in sustainable synthesis but encounters great challenges in scale-up. Photocatalysis in flow is considered as a solution for maximizing light absorption and mass transfer of reactants and thus has attracted fundamental and applied research. This perspective provides an overview of recent progress on photocatalytic chemical conversions in continuous flow from the viewpoint of system design, circulation, immobilization of photocatalysts, and solar-driven photocatalytic systems. An outlook on the future development of photocatalytic chemical conversion in flow is proposed based on a critical analysis of the challenges for applications, revealing the necessity of intensifying potentially profitable reactions using affordable photocatalysts by self-sustained automated flow systems.

## Graphical abstract

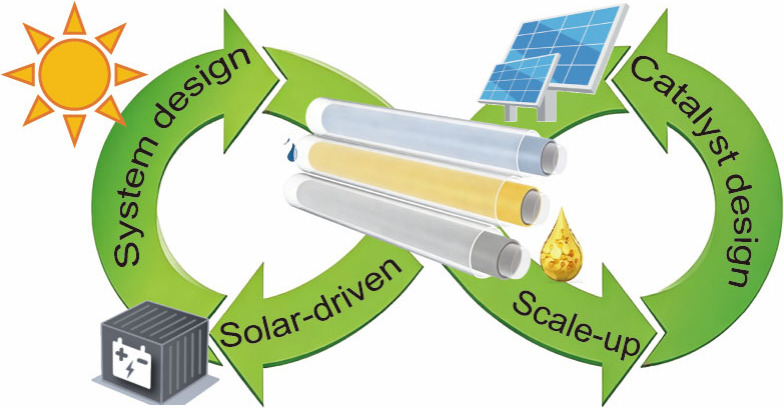

## 1 Introduction

Photocatalysis provides a solution for sustainable chemical conversions, allowing direct use of solar energy for energy storage, chemical synthesis, and pollutant decomposition.^1–3^ In recent years, a wide range of complicated and challenging redox reactions has been realized under mild conditions by employing designed homogeneous and heterogeneous photocatalysts at a lab scale,^4,5^ including hydrogen atom transfer (HAT), selective oxidation, sustainable halogenation, and cross-coupling.^6–9^ In addition, the gentle reaction conditions are ideal for late-stage conversion of pharmaceutical intermediate molecules with fragile functional groups.^10,11^ This showcases photocatalysis as a key component for circular chemistry.^12,13^ Although conventional batch reactors are simple in design,^14^ they are hampered by poor light penetration, inefficient photon–catalyst interaction, and uncontrollable residence time. This results in relatively slow reaction kinetics and limited control over selectivity in conventional bulk reaction systems,^15^ which becomes even more prominent at higher reactant concentrations.^16^ Additionally, the inefficient utilization of precious photocatalyst and organic solvents results in extra expenditure on separation, purification, and regeneration.^17–19^ Therefore, specialized reactors are necessary to facilitate the industrial implementation of photocatalysis on a practical scale.

Chemistry and catalysis in flow is featured with rapid mixing, large surface-to-volume ratio, and easy scale-up, providing unique control over reaction parameters (i.e., temperature, pressure, and concentration) to manipulate reactivity and even achieving unexpected reactions.^20–29^ The basic principles of flow chemistry with critical thinkings are presented in the excellent tutorial review “*The Hitchhiker’s Guide to Flow Chemistry*” by Plutschack and coauthors.^30^ In this masterpiece, a rationalized decision diagram clearly demonstrates that flow chemistry is (probably) the best choice for photochemistry, homogeneous photocatalysis, and heterogeneous photocatalysis, especially for light-driven synthetic chemistry that needs to be operated under complicated reaction conditions. The large surface-to-volume ratio is ideal for exposure to light, aided by employing transparent materials to construct the flow reactors. The thickness of the light absorber layer (sensitizer or photocatalyst) can be further adjusted to optimize the penetration depth of light.^31^ The nine advantages of photochemistry and photocatalysis in flow ranging from mass-transfer to safe operation are well summarized by Cambié et al.,^32^ with case studies covering organic synthesis, materials science, and water treatment. In addition, the loading of precious photocatalysts and the use of solvents can be minimized, thereby addressing issues of cost and sustainability.^33^ A wide range of reactor designs (e.g., microchannels, tubular, flat plate) have been upgraded in recent years to suit molecular photosensitizers and semiconductor photocatalysts for mono- and multi- phase reactions upon either artificial light and solar irradiations.^34–39^ Additionally, the enhanced controllability, scalability, and feasibility for the long-term operation of flow photocatalysis are the primary motivations for practical applications. However, flow systems also face technical challenges including high cost of fixed assets, risk of clogging by solid catalysts and products, and complexity for operation and maintenance. Therefore, flow reaction systems should be rationally selected and optimized case by case for amplification.

In this Perspective, we will discuss recent progress in continuous flow photocatalysis for sustainable synthetic chemistry from the selection of reaction system architectures, the photocatalysts, and the photon economy with representative case studies. The considerations and solutions of flow reaction systems for single- and multiphase reactions, including homogeneous and heterogeneous photocatalysis are presented in Section 2. The utilization and regeneration of photocatalysts in representative flow reactors via circulation and immobilization through material design and modification of reaction systems are discussed in Section 3. Based on the optimized reactor and photocatalysts, the strategies for sustainable and affordable photocatalysis via direct solar-driven chemical conversions are elaborated on in Section 4. An outlook addressing the challenges and opportunities in scale-up of photocatalytic chemical conversions is also provided, from the perspective of reactions, photocatalyst materials, and reaction systems.

## 2 System Architectures for Light-Induced Reactions

The most commonly used flow systems can be broadly categorized into microchannel and macro- (coiled) tubular systems. The selection of the flow system depends on the requirements of specific applications, with modifications based on consideration of operational parameters and protocols. These interlinked parameters include light absorption, diffusion of involved chemicals (catalysts, reactants, intermediates, and products) under dark and irradiation conditions, product separation, recycling of photocatalysts, safety, and cost. Microchannel based reactors are ideal platforms for homogeneous photochemical and photocatalytic chemical conversion owing to the superior efficiency in mixing, light penetration, and temperature control. The macro tubular reactors are more convenient for cost sensitive applications and heterogeneous systems that involve solid catalysts and gaseous reactants. The following discussion examines these architectures and their variations through selected case studies, revealing how deliberate design influences key performance metrics in flow photocatalysis.

The homogeneous systems include photochemical reactions and photocatalytic reactions employing molecular photocatalysts and photosensitizers. The most distinctive feature of homogeneous systems lies in an optimum mass transfer between light absorber and reactants,^40^ thus achieving uniform irradiation with a high efficiency. The narrow size of the microchannels ensures that the solution in flow is fully exposed to incident light, and can be further optimized to approach the penetration depth of incident light by employing the absorption coefficient and the concentration of the light responsive molecules.^41^ The progress of the reaction can be manipulated by adjusting the retention time via tuning the flow rate in a standardized continuous flow setup.^42,43^ Additionally, the microreactors typically offer significantly enhanced heat exchange capabilities compared to conventional batch glassware due to their high surface-area-to-volume ratio. Depending on the material, geometry, and flow regime, the heat transfer coefficient of a microreactor ranges from 1 to 500 MW·m^–3^·K^–1^, which is orders of magnitude higher than standard laboratory glassware (i.e., ∼10 kW·m^–3^·K^–1^ for a round-bottom flask under stirring).^44^ This ensures a minimum fluctuation in temperature for the synthesis of fragile chemicals.

Furthermore, the pressurization of microreactors is also easier than that of traditional batch systems. Jang et al. have employed a chemical vapor deposition (CVD) method to construct a parylene-based microchannel reactor by using a microfluidic mold via thermal bonding (Figure 1a).^45^ The reactor displays a high geometric surface area-to-volume ratio up to 400 cm^–1^, ensuring an enhanced efficiency of light utilization for the selective photocatalytic reduction of nitrobenzene to azoxybenzene using Eosin Y (EY) as the photocatalyst under 520 nm irradiation. The selective conversion of nitrobenzene (40 mM) and derivatives is complete within a reaction time of 50 min in a continuous flow mode under optimized reaction conditions, yet the QE has not been reported. During the reaction, triethanolamine (TEOA) is employed as the sacrificial electron donor to reduce the photoexcited EY, and thus the oxidized TEOA and the photosensitizer need to be removed after reaction. Noticeably, the productivity of this microchannel system is 4.26 nmol·h^–1^, which is an order of magnitude lower than the capillary systems, possibly due to a slow flow rate, a limited geometric area, and a low concentration of the reactant.^46,47^

**Figure 1:**
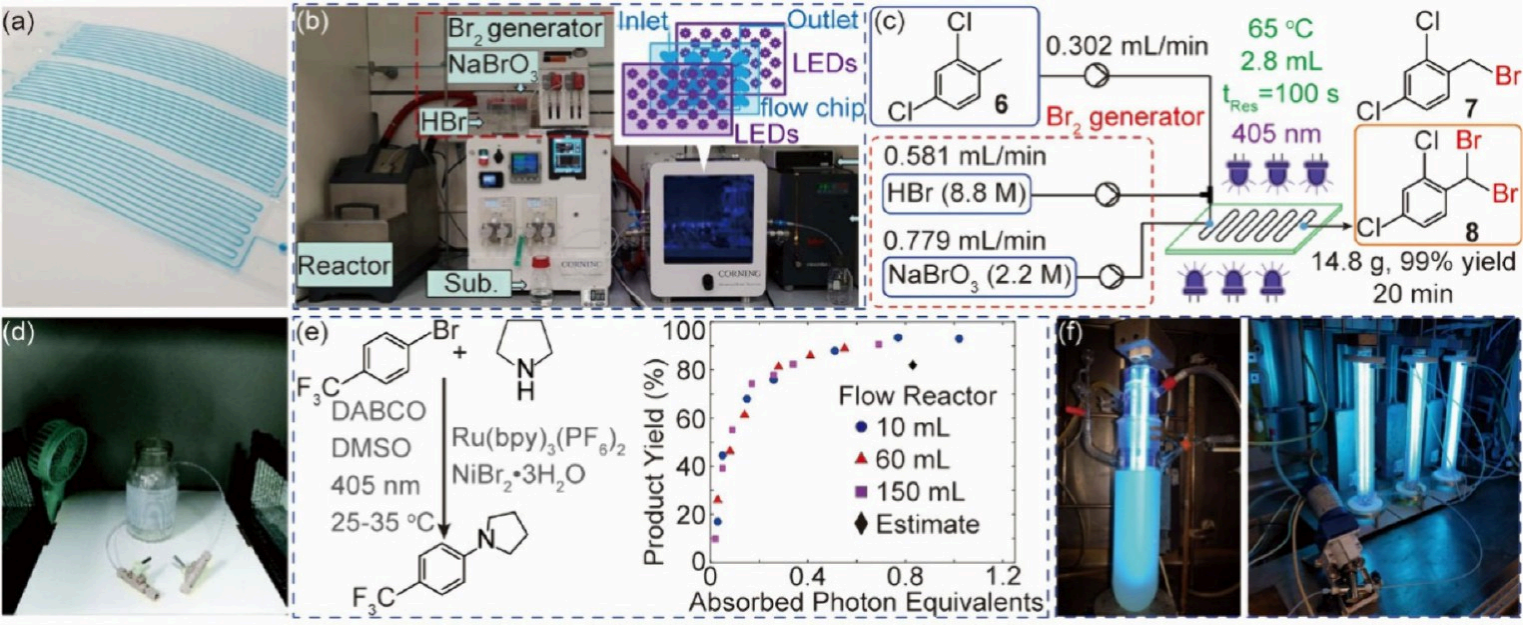
Systems for single-phase photocatalysis. (a) Microchannel reactors based on a perylene film with adjustable surface to volume ratios filled with blue dye. Reprinted with permission from ref 45. Copyright 2016, Royal Society of Chemistry.^45^ (b) and (c) Photochemical dibromination using a commercial microchannel system. Reprinted with permission from ref 48. Copyright 2019, Royal Society of Chemistry.^48^ (d) Coiled tubular reactor employing fluoropolymers for ″stop-flow″ photochemical reaction. Reprinted with permission from ref 49. Copyright 2022, Royal Society of Chemistry.^49^ (e) Reaction curves for photocatalytic C–N cross-coupling at different scales of plug flow reactors showing product yield as a function of absorbed photon equivalents. Reprinted with permission from ref 50. Copyright 2020, Wiley-VCH.^50^ (f) Photochemical decagram-scale synthesis of spirocycles based on three coiled tubular reactors in series. Reprinted with permission from ref 51. Copyright 2018, Royal Society of Chemistry.^51^

These issues have been addressed by Steiner et al. in their excellent work on continuous-flow photochemical bromination, which aims at process intensification to gain an optimum process mass intensity (PMI) and throughput.^48^ Here PMI is defined as the total mass of all materials used in a process relative to the mass of the product produced, which is a key metric used to evaluate the sustainability of chemical processes. The bromination reactions were conducted using a commercially available Advanced-Flow reactor (AFR, Corning, Figure 1b), mainly consisting of a Br_2_ generator and a photoreactor unit powered by LED. A schematic demonstration of the process is shown in Figure 1c. Elemental bromine is produced by the reaction of HBr with NaBrO_3_, which was injected into a compact glass fluidic module (G1LF) together with aromatic reactants for photochemical reactions. Both the glass fluidic module (155 × 125 × 8 mm size, 0.4 mm channel depth, 2.77 mL internal volume) and the LED are equipped with heat exchangers to control the temperature precisely. The system achieves high throughputs and reduced PMI for monobromination (1.17 kg in 230 min, PMI = 3.08) and dibromination (15 g in 20 min, PMI = 3.64) in the synthesis of representative pharmaceutical building blocks, owing to the optimized bromine generation and utilization coupled with fast interphase transfer within the microchannel photoreactor. This also leads to a high quantum efficiency (QE) of ∼43% for the synthesis of 2,4-dichlorobenzyl bromide calculated from the photon flux (690 mmol·h^–1^) of the 405 nm LED array. The same group has further upgraded this system using successive in-line modules for the metal-free photochemical synthesis of *N*-chloroamines from corresponding amines via atom-transfer radical addition.^52^ The system demonstrates remarkable stability during an operation period of 4.5 h, achieving a high throughput of 21.2 g·h^–1^ with an average yield of 94%, highlighting its exceptional process intensification capability for industrial applications. The consistently high yield achieved over a long period demonstrates the long-term operational potential of the flow system. However, there are still several issues that limit the practical applications of microchannel reactors, mainly the costs for precision manufacturing techniques (e.g., lithography, micro milling, laser processing), fixed assets (e.g., specialized materials, precision fluidic pumps, and control units), and maintenance.

The coiled capillary or tubular reactors employing fluoropolymers are the most commonly used systems due to their low cost, ease of assembly, decent optical transparency, and excellent chemical resistance to commonly used solvents, acids, and bases. Vidyacharan et al. have constructed a coiled tubular continuous flow photoreactor utilizing perfluoroalkoxyalkane (PFA) capillary tubing,^53^ enabling the direct C3-arylation of 2H-indazoles with aryldiazonium salts in the presence of EY as a sensitizer under green LEDs. The photocatalytic cycle commences with the excitation of EY to its excited state (EY*), which is quenched by a sacrificial reductant (*N,N*-diisopropylethylamine, DIPEA) via a single-electron transfer (SET) process to generate an EY anionic radical (EY^•–^) and a DIPEA cationic radical (DIPEA^•+^). Subsequently, the EY^•–^ is engaged in a second SET event with the aryldiazonium salt, reducing it to an aryl radical (Ar^·^) accompanied by nitrogen gas evolution, while regenerating the ground-state EY to complete the catalytic cycle. The C3-arylation reaction is accomplished within 1 min of green LED irradiation in a PFA capillary reactor (i_d_ = 0.25 mm) owing to a high photonic efficiency and mass transfer, which is an order-of-magnitude faster than the batch process (18 h).

In most cases, the residence time of the reaction mixture within the irradiated zones in a coiled tubular reactor is considered the key parameter to ensure complete conversion of reactants. This can be easily manipulated by running in a “stop-flow” operational mode, via integrating shut-off valves at both ends of the tubing. **Cao** et al. have constructed such a “stop-flow” coiled tubular photoreactor with PFA tubing for the direct heteroarylation of inert C–H and C–C bonds of unprotected aliphatic alcohols through visible-light-induced Minisci-type reaction in a continuous-flow mode (Figure 1d).^49^ The reaction employs phenyliodine bis­(trifluoroacetate) (PIFA) as a multifunctional reagent, which complexes with the hydroxyl group of the alcohol to generate alkoxy radicals via homolysis upon irradiation to initiate the reaction cascade. Subsequently, PIFA acts as a terminal oxidant, converting the radical adduct to the final product. The trifluoroacetic acid (TFA) produced during PIFA decomposition further protonates the heteroarene substrate, enhancing its electrophilicity and thereby facilitating the reaction with nucleophilic alkyl radicals. The stop-flow mode ensures a uniform and identical reaction time for the entire mixture once it is injected via a syringe, which eliminates the uneven residence time distribution caused by flow fluctuations.

While most works concentrate on improving the performance by controlling the residence time, Corcoran et al. establish an expression of product yield as a function of the absorbed photon equivalents for subsequent scale-up.^50^ Here, the “photon equivalents” are the ratio of absorbed photons to the reactants for a given irradiation time. This is examined using a C–N cross-coupling reaction between aryl bromides and amines in a series of PFA-based continuous-flow tubular photoreactors of varying volumes under 405 nm irradiation (Figure 1e). The reaction is initiated with a SET step from photoexcited Ru­(bpy)_3_(PF_6_)_2_ to a Ni­(II) precatalyst, generating a highly active Ni­(I) intermediate that subsequently undergoes oxidative addition into the C–Br bond of the aryl bromide to form a Ni­(III) complex. The Ni­(III) complex interacts with an amine via transmetalation and reductive elimination to generate a C–N bond while regenerating the Ni­(I) catalyst. Concurrently, the Ru­(bpy)_3_^3+^ species accepts an electron during the reductive elimination step, returning to its ground state Ru­(bpy)_3_^2+^ to complete the catalytic cycle. Remarkably, the product yield– absorbed photon equivalent plots at various reactor volumes up to 3.5 L (preparatory scale) are almost overlaid, implying that the absorbed photon equivalents are a reliable empirical parameter for precise scaling-up. This reaction scales seamlessly from microliters to 3.5 L while maintaining high efficiency, demonstrating the superior scalability of the flow platform.

Elliott et al. show that three challenging photochemical reactions, including the Norrish-Yang cyclization of *N*-substituted succinimides, the rearrangement/ring-opening of pyridinium salts, and the cyclization of pyrroles, can be realized at a decagram-scale using a compact coil reactor with a centered 36 W UVC low-pressure mercury lamp (Figure 1f).^51^ Here fluorinated ethylene propylene (FEP) tubing is employed due to its excellent transparency in UV (particularly UVC) and chemical inertness. The FEP tubing (2.7 mm ID/3.1 mm OD) was coiled around a quartz tube (360 mm length/48 mm OD) housing a 36 W UV–C germicidal lamp (PL-L, Philips), with both ends sealed by using polytetrafluoroethylene (PTFE) caps. A scaled reactor containing three modules in series with a total volume of ∼270 mL is constructed, enabling the Norrish-Yang cyclization and the synthesis of functionalized spirocycles at a productivity of 4 g·h^–1^ and 0.46 g·h^–1^, respectively, which are ∼5–6 times higher than the batch system. The scale-up of the continuous coiled flow system has been successfully employed by Bottecchia et al. for light-induced production of an anticancer drug (Belzutifan) at a daily production rate of ∼100 kg·via bromination, which has received the good manufacturing practices (GMP) certification.^54^ The production capacity of the FEP tubing based flow reactor is rationally scaled by connecting multiple identical modules in parallel, based on previously mentioned “photon equivalents” as a geometric- independent scaling parameter. The major challenge of FEP reactors for practical application is the reduced light penetration and risk of tubing blockage caused by a limited solubility of the product, especially at a higher concentration of reactants. Horie et al. have discovered that insoluble photodimerization products of maleic anhydride (MA) can clog a conventional microreactor.^55^ This problem can be solved by gas disturbance combined with sonication, enabling continuous operation of the slug flow for more than 16 h without clogging.

Nevertheless, a careful selection of the tubing material is essential for the coiled tubular system. Presently, the most commonly employed fluoropolymers are PFA, FEP, and PTFE. While the PTFE tubing exhibits excellent chemical and mechanical durability, the shape memory effect requires delicate temperature control during operation to prevent leakage caused by thermal recovery. The relatively poor optical transparency of PTFE also limits its applications. Both PFA and FEP demonstrate exceptionally high transmittance across the UVA and visible spectral regions (>95%), though PFA is preferred for UVB and UVC irradiation, owing to a shorter UV-cutoff wavelength. Some critical parameters of frequently used tubing materials for photocatalysis are available in our recent perspective.^56^

The introduction of gas phases into the liquid phase can be relatively easily realized in the aforementioned microchannel reactors. Representatively, Wu and coauthors have examined the hydrodynamics of single- and two-phase flow in the G1 fluidic module of the Coring Advanced-Flow photoreactor.^57^ The microfluidic chip is featured with multiple inlets and a patented “HEART” structure, aiming at simultaneous introduction and instant mixing of gaseous and liquid reactants. The excellent transparency within 300–800 nm allows the use of particle image velocimetry (PIV) for optical characterization of the flow field for both single-phase flow (water) and two-phase flow (nitrogen + water). It can be clearly seen that the two-phase flow experiences a splitting-recombining process when entering the heart cell (Figure 2a), which changes the direction of the velocity for improved mixing. It is found that the average bubble size and the bubble size distribution decrease with the reduction of the gas volume transport fraction at a fixed overall flow rate (30 mL·min^–1^). A computational fluid dynamics (CFD) analysis of the velocity field reveals that increasing the gas volume transport fraction barely influences the velocity magnitude, but tunes the velocity direction. A comparative vector plot of the velocity field in single-phase (green) and two-phase (red) flow clearly visualizes the effect of gas bubbles (Figure 2b), which forces the liquid phase in the upper half of the heart cell to flow downward and the lower half to flow upward. This results in an increase in the local velocities in the region between the two obstacles, thus reducing the stagnant fluid zone observed for the single-phase flow, in which the fluid travels mainly along the reactor wall, creating a large U-shaped stagnant flow region. The effect of two-phase flow on momentum exchange was further quantified by addressing the components and computing the root-mean-square (RMS) velocity fluctuation. Interestingly, introducing a gas phase does not increase the magnitude of average RMS velocity fluctuation but reduces its standard deviation, implying a more homogeneous mixing for the two-phase flow than the single-phase flow within the cell.

**Figure 2:**
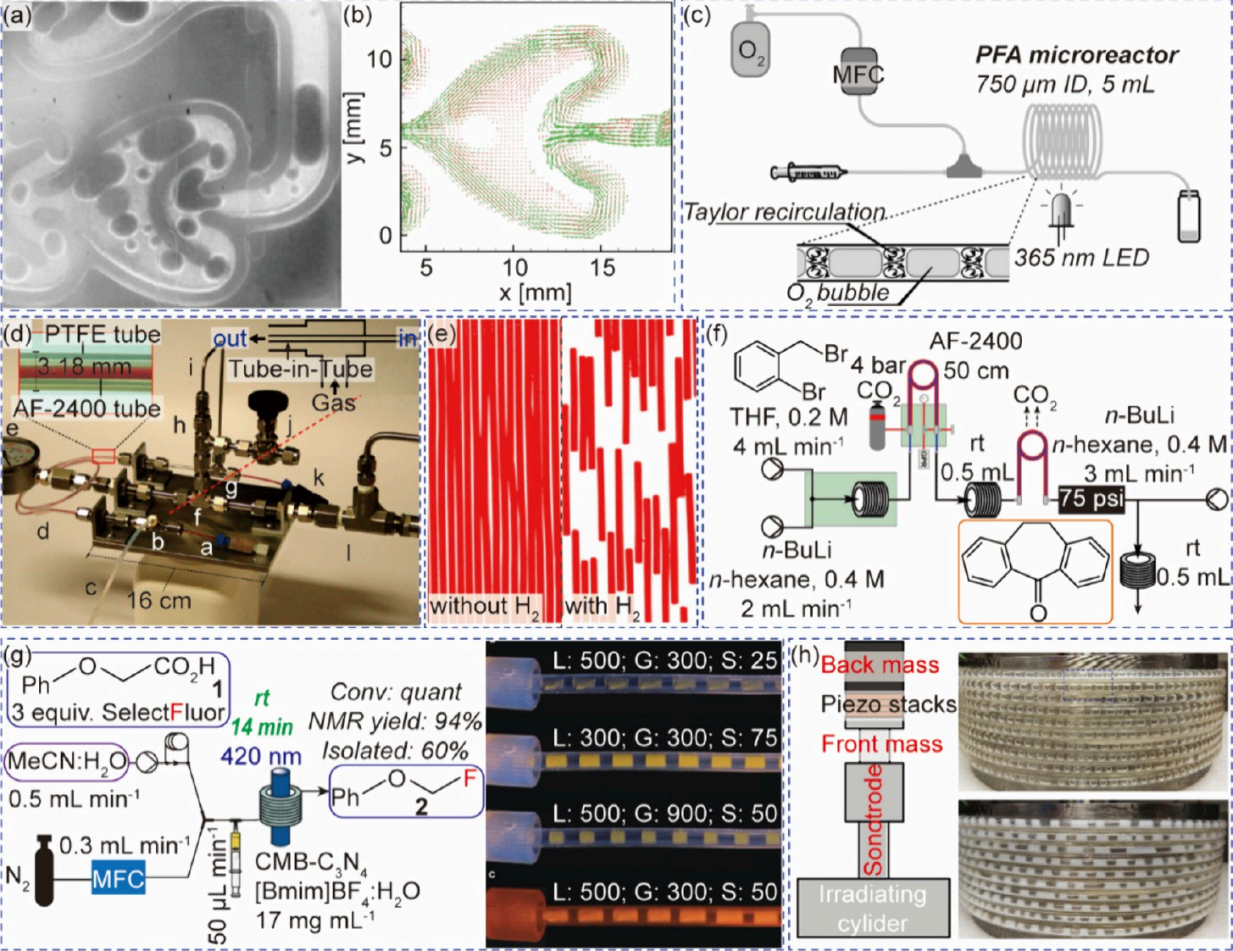
Systems for multiphase photocatalysis. (a) Bubbles in “heart” of the Corning AFR module. (b) Velocity vector fields of single-phase flow (green vectors) and two-phase flow (red vectors) in the heart-shaped cell. Reprinted with permission from ref 57. Copyright 2015, American Chemical Society.^57^ (c) Continuous flow setup for gas–liquid reaction in a segmented flow regime. Reprinted with permission from ref 61. Copyright 2018, Wiley-VCH.^61^ (d) A prototype tube-in-tube reactor (e) Bubble counting of outgassed reaction stream for quantitative optimization of hydrogen flow. Reprinted with permission from ref 59. Copyright 2015, American Chemical Society.^59^ (f) Stage one of a multistep tube-in-tube flow for the synthesis of amitriptyline. Reprinted with permission from ref 65. Copyright 2013, Wiley-VCH.^65^ (g) A triple-phase continuous flow system with adjustable ratios of gas and suspension segments. Reprinted with permission from ref 66. Copyright 2018, Wiley-VCH.^66^ (h) Mesoscale ultrasonic milli-reactor for triple-phase continuous flow reaction. Reprinted with permission from ref 67. Copyright 2022, Elsevier.^67^

Mandigma et al. have employed such a system for the late-stage N–CH_3_ selective photocatalytic oxidation of trialkylamines to *N*-formamides in continuous flow, using molecular oxygen as the oxidant.^58^ The reaction layer of the reactor is sandwiched between two heat transfer layers, allowing for precise temperature control. In addition, a back-pressure regulator is applied prior to the collector, which upgrades the heterogeneous slug flow into a homogeneous flow to increase the solubility of O_2_. The system enables the synthesis of a series of complicated *N*-formamides with reasonable yields at optimized irradiation, residence time, pressure, and temperature, which is more efficient than that in a commercial tubular coil continuous flow photoreactor. The authors smartly functionalize the original 9,10-dicyanoanthracene (DCA) photocatalyst with sulfonamide groups to improve its solubility in polar aprotic solvents, which is essential for the operation in microchannel and even tubular flow reactors. However, it must also be considered that the extra synthetic steps involved in functionalization results in extra effort and cost for scaling-up applications.

The gas–liquid systems can also be upgraded from the coiled reactor configurations, with the focus on enhancing the gas–liquid mass transfer efficiency. Simply introducing two fluid streams into the reactor may result in inhomogeneous gas–liquid mixtures or stratified flow.^59^ Incorporating a T-mixer at the point of stream convergence enables intense collision and shearing between the gas and liquid phases at optimal flow rates, which pinches off the continuous gas phase into uniform bubbles that are segmented by the liquid phase.^60^ The gas–liquid streams may turn into Taylor flow at certain velocity of the fluids, where the increased interfacial area and the axisymmetric toroidal vortices of individual fluid facilitate efficient mass transfer of chemicals. This facilitates reactants from the slug core approaching the gas–liquid interface for the reaction and removal of products from the interface simultaneously. Laudadio et al. report the tetrabutylammonium decatungstate (TBADT) photocatalyzed aerobic oxidation of C­(sp^3^)–H bonds in cycloalkanes with molecular oxygen as the oxidant in a PFA based microreactor under a segmented flow regime (Figure 2c).^61^ This is realized by mixing the injected liquid phase via a syringe pump and dosed gaseous oxygen through a mass flow controller (MFC) in a T-mixer prior to the coiled reactor. Note that the use of a back-pressure regulator (5.2 bar) is crucial to maximize the solubility of oxygen under atmospheric pressure to achieve an optimum yield of desired product. The space-time yield in flow is increased by an order of magnitude, reflecting the advantages of flow systems in enhancing mass transfer and process intensification.^62^ The reaction is initiated via a hydrogen atom transfer (HAT) process between the photoexcited TBADT and reactant, generating a pivotal alkyl radical (^•^R), which is subsequently trapped by molecular oxygen to initiate a cascade of follow-up reactions. The same group has also detailed the protocols for the construction of a photochemical microreactor for gas–liquid photoredox catalysis, with the representative synthesis of disulfides and trifluoromethylated compounds as case studies.^63^ The modular designed reactor is assembled with commercially available parts, featuring it as an economic solution for efficient photocatalysis employing a wide range of homogeneous photocatalysts (e.g., Ru­(bpy)_3_ and EY). Lévesque et al. has developed a single, fully integrated continuous-flow system for the synthesis of artemisinin at a daily production rate of 200 g.^64^ The system consists of a flow photochemical reactor for the photooxidation of dihydroartemisinic acid with tetraphenylporphyrin (TPP) as the photosensitizer under O_2_ atmosphere, and a commercially available flow system for further Hock cleavage and oxidation via triplet oxygen, thus eliminating separation and purification of intermediates.

Yu et al. present the photocatalytic synthesis of hydrogen peroxide (H_2_O_2_) from oxygen reduction in flow using a similar apparatus,^68^ using glass beads (d = 0.5 mm) packed into the PFA tubing for better mass transfer in a reduced dead volume. Additionally, a triangular pyramid-shaped reflector is placed in the bottom-middle of the coiled tubular reactor, aiming at the efficient utilization of the incident light. The photocatalytic synthesis of H_2_O_2_ is initiated by using 2-ethylanthraquinone (EAQ) as a photosensitizer and alcohol as the electron donor at 50 °C under 0.5–1.0 MPa of O_2_. It is reported that such continuous-flow system shows a remarkable 91-fold enhancement in H_2_O_2_ production rate than the conventional batch reactor, and can be connected with a liquid–liquid extraction device to realize continuous separation of H_2_O_2_ in aqueous solution of up to 11.1 wt % in 1 h. The system also depicts a lower loading of EAQ (0.2–0.3 mol %) than the traditional industrial anthraquinone process (AO process).

It is also convenient to introduce gases into the reaction by employing a tube-in-tube design, using the chemically stable Teflon AF 2400 fluoropolymer tubing, which displays exceptional permeabilities of many gas molecules (e.g., CO_2_, O_2_, H_2_, and N_2_) that is 2–3 orders greater than the PTFE tubing.^59^ A prototype tube-in-tube reactor is shown in Figure 2d, where the Teflon AF-2400 tubing is fixed within PTFE tubing with a larger diameter. The inner and outer tubing are separated by Swagelok T-pieces to avoid direct contact. The tube-in-tube system primarily functions as a premixing zone for gas and liquid phases, rather than serving as the reaction site. In most common configurations, the gas flows through the outer tube while the liquid passes through the inner tube. During operation, a significant gas concentration gradient forms across the Teflon AF-2400 tubing. According to Henry’s law and Fick’s law of diffusion, gas molecules spontaneously migrate from the high-concentration side to the low-concentration side, ultimately diffusing into the liquid stream. This process achieves molecular-level dispersion, forming a homogeneous gas–liquid solution. A back-pressure regulator installed at the outlet maintains system pressure, preventing gas from precipitating out of the solution due to sudden pressure drop and thereby ensuring uniformity in the liquid-phase reaction. The gas-saturated homogeneous solvent flows into the coiled reactor for further chemical reactions. The tube-in-tube reactor represents a significant advancement for handling gaseous reagents in flow. A minimal volume (1–2 mL) of pressurized gas at any given time minimizes the risks of using toxic and flammable gases (e.g., Cl_2_, CO, and H_2_). O’Brien et al. have realized complete hydrogenation of alkenes within 93 s, underscoring the rapid and efficient gas–liquid mixing in the tube-in-tube reactor.^69^

Several methods have been developed for the precise quantification and control of the inlet gas reactants in the tube-in-tube reactor. Titration is a convenient method for the determination of acidic and alkaline gases (e.g., CO_2_, NH_3_, and H_2_S).^70^ For gases with characteristic infrared vibrational peaks, quantitative Fourier transform infrared (FTIR) spectrometry can be employed in real time to monitor and control the concentration of dissolved gas in the liquid stream (e.g., CO and CO_2_).^71^ O’Brien and coauthors have developed a computer-based real-time “bubble counting” technique for the quantification of insoluble gases (Figure 2e), by using a digital camera at the outlet of the tube-in-tube reactor.^69^ Here, the supersaturated homogeneous liquid turns into a segmented flow due to the pressure drop. By comparing a H_2_-pressurized flow (experimental run) with a nonpressurized flow (calibration run), the quantity of H_2_ after reaction can be quantified by analyzing the ratio of white pixels (bubbles) to total pixels in the digitalized imaging. A dye can be added to the liquid to increase the contrast. The original concentration of dissolved H_2_ can therefore be estimated using the inlet and outlet volumes, thus achieving an intelligent hydrogenation of alkenes in the tube-in-tube system.

A two-stage tube-in-tube system provides an effective strategy for removing generated gas product(s) and excess gas reactant(s) introduced in the first stage by applying a reduced pressure to the secondary tube-in-tube unit. This deliberate pressure control promotes the permeation of gases out of the liquid reaction stream, thereby shifting the reaction equilibrium and simplifying the downstream processing. Krischning et al. have constructed a two-stage multistep flow system for the synthesis of tricyclic antidepressant amitriptyline, employing two tube-in-tube reactors in the first stage (Figure 2f).^65^ The first step in Stage one employs a tube-in-tube reactor for the introduction of CO_2_ for cyclization of 2-bromobenzyl bromide at −50 °C, followed by a lithiation of the generated intermediate after a second tube-in-tube device for the removal of excessive CO_2_ at a reduced pressure at room temperature (RT). A high isolation yield of ketone (76%) is achieved in an overall residence time of ∼33 s, which is significantly efficient compared with the batch procedure (38–56% yield, 2 h) that operates at −100 °C.

Kouridaki and Huvaere have realized the photocatalytic oxidation of ethyl 3-(2-furyl)­propanoate with molecular oxygen in a tube-in-tube system.^72^ The enclosed and pressurized reactor system effectively minimizes risks by preventing the formation of flammable solvent vapors under an oxygen atmosphere. The aforementioned “bubble counting″ method has been adopted to quantify the solubility of oxygen in different solvents (MeOH, MeCN, and DCM) and at various temperatures (25, 50, and 110 °C). An optimum productivity of 2.75 mmol·h^–1^ and a space time yield (STY) of 1.4 mol·L^–1^·h^–1^ are achieved employing methanol as the solvent at 110 °C. A remarkable isolated yield (90%) is obtained within 5 min of irradiation at 70 °C, which is significantly enhanced compared with the segmented flow system (65% conversion for 2.5 min of irradiation). The productivity is strongly correlated to the concentration of reactants, which peaks at 50–100 mM but drops at 200 mM, possibly due to an insufficient light exposure. The performance can be enhanced by further increasing the oxygen pressure and reaction temperature, yet the risk on safety needs to be considered. The scaling-up of the tube-in-tube reactor to a practical level is the major challenge for applications. A computational modeling by Jensen et al. reveals the failure in reaching a high throughput by simply increasing the diameter of the tubing, due to a drop in gas saturation fraction caused by a gradient-driven diffusion.^73^ Parallelization of membrane units can maintain the catalytic performance at elevated flow rates (e.g., ∼50 mL·min^–1^), but escalating complexity and cost limit its economic viability for large-scale catalysis.

For multiphase reactions involving solids (i.e., catalysts), flushing all chemicals into the flow system is a straightforward method but also could be a problematic solution. It is very seldomly used for microchannel reactors due to the risk of clogging. Immobilization of the solid photocatalysts inside the microchannels for heterogeneous photocatalysis will be discussed in detail in the following section. It is possible to inject solid-containing suspensions for tubular systems with larger diameters. Generally, a smaller particle size of photocatalyst improves the mass transfer but results in complicated separation, whereas a larger particle size of photocatalyst may reduce the loss of catalyst during operation but improves the risk of clogging.^74^ Fluid dynamics is another key factor that influences the reaction process. While laminar flow may lead to a poor mixing and channeling, turbulent flow promotes an enhanced homogeneity of the flow and irradiation but may induce loss of photocatalyst.^75,76^ Li et al. shows that a mixture of photocatalyst (hollow conjugated microporous polymer nanotubes) and liquid reactants (*N*,*N*- dimethylacrylamide, DMA) can be directly injected into a FEP tubular (d = 2 mm) continuous-flow photoreactor for polymerization reactions via a photoinduced electron/energy transfer-reversible addition–fragmentation chain-transfer (PET-RAFT).^77^ A high conversion of DMA (>90%) and a narrow dispersity (∼1.1) of the polymerized DMA is achieved at an initial DMA concentration of 5.0 M and a flow rate of 15 μL·min^–1^ under yellow light irradiation (λ_max_ = 570 nm, 3.6 mW·cm^–2^) within 44 h. This system shows a decent versatility for the polymerization of acrylamides and acrylates and high durability in cycle tests, possibly due to a relatively low loading of photocatalyst and a high dispersion in dimethyl sulfoxide (DMSO) that avoids the agglomeration of the photocatalyst powders.

Pieber et al. show that dosing a photocatalyst suspension into a segmented gas–liquid flow provides an alternative solution for heterogeneous photocatalytic decarboxylative fluorination reaction **(** Figure 2g).^66^ The system consists of a gas–liquid inlet for the generation of a stable N_2_–reactant solution segmented-flow using a Y-shaped mixer, a suspension inlet to dose carbon nitride photocatalyst via a vertically mounted syringe pump that incorporates a magnetic stir bar through a T-mixer, and a coiled tubular reactor for irradiation. A fine control over the segmented-flow is realized by manipulating the flow rates of individual substances. Here, viscous solvents (i.e., 1-butyl-3-methylimidazolium tetrafluoroborate ([Bmim]­BF_4_) and ethylene glycol) are preferred to form a stable photocatalyst suspension for the accurate dosing of solid materials. The flow rates of the gas, reactant solution, and catalyst suspension can be manipulated to form uniform triphasic flow, which resembles a series of small solid–liquid batch reactors that are separated by an inert gas spacer and “stirred” by toroidal flows. This flow pattern also induces strong internal circulating vortices within each liquid slug, effectively promoting a uniform dispersion of catalyst particles and suppressing their deposition and adhesion to the tube wall. Additionally, the generated CO_2_ can readily diffuse into the pre-existing gas segments during the reaction, achieving “*in situ* degassing” that effectively mitigates pressure buildup and impaired mass transfer caused by gas accumulation. Since the light scattering and absorption in gas are much lower than in the suspension, the system also presents enhanced efficiency in photon utilization. These features lead to efficient and selective photocatalytic fluorination of a wide range of chemicals compared with traditional batch reactors. Furthermore, the process can be easily scaled up for the gram-scale synthesis of a monofluorinated compound using a setup with two catalyst addition units. Shang et al. have utilized this type of flow system for photocatalytic remediation of antibiotics wastewater using a Bi_2_WO_6_ photocatalyst,^78^ which shows a 1.6 times enhancement in apparent reaction rate constants than the batch counterpart. The mass transfer and oxygen enrichment can be adjusted by tuning the length of suspension and gas segments, thus achieving an optimum photocatalytic performance. High-speed imaging analysis reveals that the solid photocatalyst particles exhibit a regular, toroidal flow trajectory at the bottom of the suspension slugs during the reaction. Furthermore, a relatively small deviation between the experimentally and ideal recovered mass of photocatalyst (<4.5%) on the outlet stream confirms the uniform distribution of the photocatalyst suspension in the flow system.

Dong et al. have constructed a mesoscale ultrasonic milli-reactor that consists of a Langevin-type transducer, a sonotrode, and an irradiating cylinder (attached with a 12.88 mL coiled glass capillary, Figure 2h).^67^ The cavitation bubbles generated by sonication result in a vigorous oscillation that improves the mixing of liquid and gas–liquid mass transfer. More importantly, the settlement of TiO_2_ photocatalyst particles can also be minimized, thus avoiding the clogging of the reaction channels. The vibration efficiency is maximized by combining longitudinal resonance transmission with radial resonance conversion through the synergistic effect of the component structures. The longitudinal vibration generated by the transducer is transmitted via the sonotrode to the cylinder, which is then converted into a radial vibration by the cylinder, a radial half-wavelength resonator. This special structure generates an intense and uniform ultrasonic field within the capillary coiled around the cylinder. The precipitated TiO_2_ particles form a homogeneous emulsion-like suspension upon applying sonication, demonstrating the efficiency and reversibility of the system. The selective photooxidation of *p*-trifluoromethylbenzyl alcohol (1–5 mM) to *p*-trifluoromethylbenzaldehyde catalyzed by TiO_2_ is realized under an oxygen atmosphere and 365 nm LED irradiation, achieving a 4-time increase in conversion compared to the reactor without sonication. Notably, a pulsed sonication with active cooling is necessary for reactions that are sensitive to temperature increase. Similarly, Rosso et al. has employed a commercially available plug flow photoreactor (HANU) for the synthesis of tetracaine at a gram-scale with a rate of 2.67 g·h^–1^.^79^ The fluid exhibits a near-plug flow behavior by precisely tuning the oscillation amplitude, frequency, and injection rate of argon, enabling a stable and continuous operation for over 5 h. The characteristics of flow systems are summarized in Table 1, and a decision diagram is provided to facilitate the selection of reactors for specific photocatalytic reactions (Scheme 1). The immobilization, separation, and circulation of photocatalysts in flow systems are discussed in detail in section 3.

**Table 1:** Comparison of Flow Photoreactors and Their Applications

Phase	Reactor	Pros and *Cons*	Examples
Single phase	Microchannel	Mixing; Uniform irradiation; Temperature control	Hydrogenation
		*Cost; Complexity; Maintenance; Clogging*	Arylation
			
	Coiled tubular	Cost; Assembly; Chemical resistance	C–N coupling
		*Mass transfer; Ununiform irradiation; Clogging*	
			
Gas–Liquid	Coiled tubular with T-mixer	Mixing; Assembly	Oxidation
		*Unstable flow pattern; Gas flow rate sensitive*	H_2_O_2_ synthesis
			
	Microchannel	Mixing; Uniform irradiation; Temperature control	Oxidation
		*Cost; Complexity; Maintenance*	
			
	Tube-in-tube	Gas dispersion; In-line gas monitoring; Safety	Hydrogenation
		*Cost; Complexity*	CO_2_ cyclization
			Oxidation
			
Solid–Liquid	Coiled tubular	Slurries; Assembly	Polymerization
		*Catalyst precipitation; Light penetration*	
			
	Packed-bed	Catalyst preservation; Continuous operation	Halogenation
	Fixed-bed	*Mass transfer; Light penetration*	N–N coupling
			
Triphase	Segmented flow	Catalyst dispersion; In-situ gas exchange	Fluorination
		*Complexity; Catalyst loss*	Oxidation

**Scheme 1:**
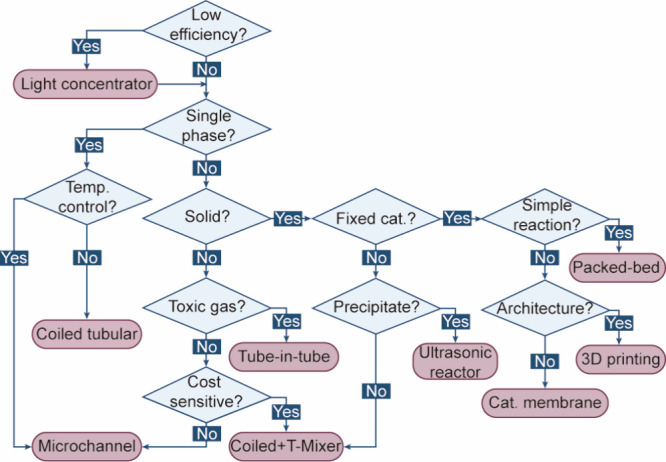
Decision Diagram for Photocatalysis in Flow

## 3 Photocatalysts: Circulation vs Immobilization

The effective management of photocatalysts is essential for realizing sustainable and scalable flow processes. This section discusses strategies for catalyst reuse, recovery, and integration within flow systems. The approaches for recycling and reuse of homogeneous photocatalysts include heterogenization, membrane-based separation, scavenging methods, and biphasic systems. The immobilization of heterogeneous photocatalysts can be realized by packing, coating, and 3D printing of structured catalysts. The impacts on catalyst leaching, light absorption, mass transfer, and system durability will be covered.

The separation and recycling of homogeneous transition metal catalysts in continuous flow systems include heterogenization, scavenging, and filtration, which have been systematically summarized by Gürsel et al.^80^ The heterogenization of homogeneous photocatalysts can be realized by polymerization,^81^ anchoring on supports,^82^ and crystallization,^83^ forming heterogeneous photocatalysts exhibiting porous structures or with tunable solubility in different solvents to maximize light absorption and mass transfer. However, very few works employ the heterogenized photocatalyst in flow. Rossi et al. have immobilized EY on a Merrifield resin (MR) for direct arylation of furan with aryldiazonium salts under green light irradiation.^84^ The Merrifield resin, typically available as white beads, is a cross-linked polystyrene resin that consists of a chloromethyl functional group. The material swells in solvents (e.g., ethyl acetate, DMF, and DMSO), allowing nucleophilic addition of EY in its inactive hydrogenated form with the presence of *N,N*-diisopropylethylamine (DIPEA) at 80 °C for 72 h under stirring, forming the MR-EY heterogeneous photocatalyst at a 20 g scale (Figure 3a). The heterogenized MR-EY photocatalyst shows a comparable yield of alkylated arene compound (52%) with the molecular EY photosensitizer in a batch reactor (55%), suggesting that the anchoring process barely influences the intrinsic properties of EY. A packed-bed reactor filled with the MR-EY photocatalyst and glass beads is constructed for photocatalytic arylation in flow, providing a reasonable product yield of 56% at a residence time of 10 min (150 μL·min^–1^). The productivity (0.89 mmol·h^–1^) and the STY are ∼2.5 and 8.8 times higher than those of the batch system, demonstrating the efficiency of the flow system; however, a rapid decay in performance is observed for the second and third runs, possibly due to the destruction of the catalyst. Therefore, the interaction of the photocatalyst with the support should consider the electronic structure of the material. A theoretical investigation by De Vos and coauthors explores the anchoring of Ru­(II) polypyridyl complexes onto covalent triazine frameworks (CTF) with tunable content of nitrogen.^85^ They show that the band gaps, charge transfer reactions, and redox potentials of the system are adjustable by tuning the nitrogen content. A higher nitrogen content lowers the energy of unoccupied polypyridyl levels and occupied Ru t_2g_ levels, thus favoring an enhanced light-induced charge transfer. Additionally, the stability of the heterogenized photocatalyst is solely dependent on the linker, implying wide applicability for the immobilization of the Ru­(II) complex on MOFs. However, the heterogenization process may not be appropriate for certain reactions that require significant redox changes in the catalyst. A specific case is the Pd-catalyzed cross-coupling, where Pd^0^ species need to transform into soluble Pd^2+^ active species during the reaction,^86^ thus leading to the leaching of Pd from the support and a reduced activity.

**Figure 3:**
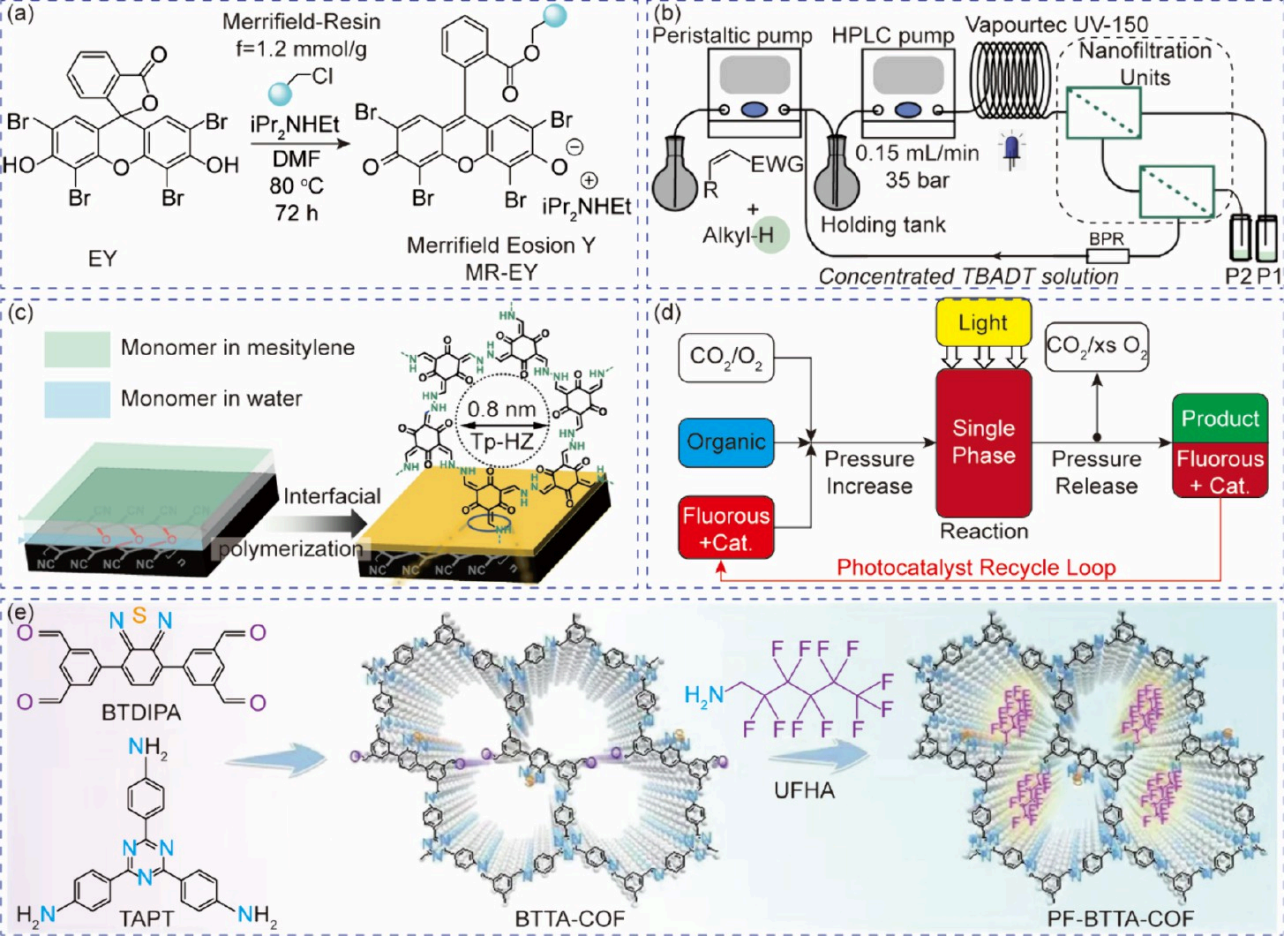
Separation and recycling of molecular photocatalysts. (a) Heterogenization process of the EY. Reprinted with permission from ref 84. Copyright 2022, MDPI.^84^ (b) A multistage nanofiltration-based continuous-flow system for the recovery of TBADT sensitizer. Reprinted with permission from ref 87. Copyright 2022, Springer Nature.^87^ (c) Preparation procedures and chemical structures of COF membranes for the separation of photocatalysts and products. Reprinted with permission from ref 88. Copyright 2023, Springer Nature.^88^ (d) Continuous photo-oxidation reaction in a scCO_2_-fluorous solvent system. Reprinted with permission from ref 89. Copyright 2012, Royal Society of Chemistry.^89^ (e) Synthetic procedure of a hydrophobic PF-BTTA-COF photocatalyst for H_2_O_2_ production in a biphasic system. Reprinted with permission from ref 90. Copyright 2024, Springer Nature.^90^

Scavenging is an alternative solution for the separation and recycling of homogeneous photocatalysts with noble metals, which extracts metals by forming insoluble metal-chelate complexes using scavengers.^91^ This process can be realized by flowing the catalyst through columns that contain solid-supported scavenging resins or directly applying scavenging agents in solution.^92^ Despite their efficacy in processing up to gram-scale, larger-scale operations of the columns are rare due to their limited capacity.^93^ Frequently employed water-soluble scavenger agents include ethylene diamine tetraacetic acid (EDTA), diethylene triamine pentaacetic acid (DTPA), and ethylene diamine disuccinic acid (EDDS).^94,95^ Gürsel et al. have embedded a flow separator with a porous fluoropolymer membrane for online liquid–liquid extraction of Cu after the azide–alkyne cycloaddition (CuAAC) click reaction in flow.^96^ A high yield of the triazole product (up to 92%) with pharmaceutical purity can be achieved through a single separation stage by fine-tuning the scavenger type, quantity, and pH. The scavenging method should be applicable for photocatalysis in flow, yet has not been reported to the best of our knowledge.

Employing a membrane for the filtration of homogeneous catalysts by molecular weight by applying pressure is a straightforward solution that can be adopted for photocatalytic chemical conversions. Organic solvent nanofiltration (OSN) based on inert membranes is considered as a sustainable approach due to a low energy consumption.^97–99^ The molecular weight cutoff (MWCO) of specific membranes enables chemicals with smaller molecular weight (including product, reactants, and solvent) to pass through while catalysts with larger molecular weight are retained. The so-called “molecular weight enlargement (MWE)” method can be used to improve the separation efficiency of homogeneous catalysts with comparable molar mass to the reactants and products.^100^ This is realized by attaching homogeneous catalysts to soluble supports, including dendrimers, polymers, and polyhedral oligomeric silsesquioxanes (POSS).^101^ The continuous membrane reactor can be operated in a dead-end filtration mode or a loop mode. Vankelecom et al. have summarized the membrane materials, preparation methods, and transport mechanisms.^102^ A two stage OSN has been successfully embedded with a Vaportec UV-150 flow reactor by Timothy et al. for continuous flow photocatalytic C­(sp^3^)–H alkylation, employing a tetrabutylammonium decatungstate (TBADT) photocatalyst in an acetonitrile solution (Figure 3b).^87^ In this case, the major consideration for membrane selection is their stability in harsh solvents (acetonitrile and acetone), due to the significant difference in molecular weights of TBADT (3320 Da) and reagents (<400 Da). The screening of five commercial membranes by measuring the flux and selectivity during filtration of a TBADT-acetonitrile solution (1 mM) reveals that the NF080105 and NF030306 membranes from SolSep BV withstand filtration in acetonitrile and exhibit a decent rejection of TBADT.^103^ The NF030306 membrane is the optimum candidate owing to better stability at prolonged running time under higher pressures, allowing the system to be operated at a relatively high pressure (35 bar) and a flow rate of 0.15 mL·min^–1^ without precipitation of the photocatalyst. Innovatively, a two-stage OSN is applied for this system to increase the overall permeated volume and product recovery. A lower flow rate in the second OSN results in equilibrium flux values lower than the first stage, achieving a catalyst retention that exceeds 99% and an overall catalyst recovery of 98.4%, paired with a satisfactory product recovery exceeding 80%. Remarkably, the fraction yield of photocatalytic alkylation product remains high and stable throughout a 3-day continuous running with a total TON of 6738. Although some precipitation of TBADT is observed on the membrane, it could be removed via periodical backwashing with acetonitrile. Additionally, this system is well-adapted to other photocatalysts with smaller molar masses, including 4CzIPN, Ru­(bpy)_3_(PF_6_)_2_, and Ir­[(dF­(CF_3_)­ppy)]_2_(dtbpy))­PF_6_), although slightly reduced recovery rates are observed.

Although the selection pool of polymeric membranes for the OSN is huge, the limited porosity and poor tolerance to polar solvents restrict their applications. Yang et al. have developed a method to grow thin covalent organic framework (COF) membranes with tunable pore size on a carbonized polyacrylonitrile (PAN) substrate through an interfacial polymerization process (Figure 3c).^88^ The original PAN membrane is first carbonized under an inert atmosphere with the addition of a pore-forming agent, [Ca­(NO)_2_], to yield a chemical resistance substrate with increased porosity. The resulting carbonized PAN substrates display favorable stability in dimethylformamide (DMF), 1-methyl-2-pyrrolidone (NMP), and dimethyl sulfoxide (DMSO) for 60 days, with a decreased tensile strength and enhanced Young’s modulus. The two-dimensional imine-linked COF membranes with a thickness of ∼100 nm are then synthesized *in situ* on the carbonized PAN substrates through an interfacial polymerization process, by a sequential dosing of water-soluble amine monomers and mesitylene-soluble aldehyde monomer (1,3,5-triformylphloroglucinol, Tp) following a dehydrative coupling in an acetic acid aqueous solution at 60 °C for 36 h. The performance of COF membranes for recycling metal-based photocatalysts is evaluated using a series of well-established Ir- and Ru- based photoredox catalysts for hydrogen atom transfer (HAT), energy transfer (EnT), metallaphotoredox, and enantioselective reactions with the presence of a wide range of solvents, including *N,N*-dimethylacetamide­(DMA), hexafluoroisopropanol (HFIP), dichloromethane (DCM), methanol, DMSO, acetonitrile, tetrahydrofuran (THF), and 1,4-dioxane. The tunable pore size of the COF membrane allows the optimization of rejection rates and permeance for specific photocatalysts. Remarkably, photocatalytic C–H fluorination is realized in a continuous flow reactor on a 90 mmol-scale using a sodium decatungstate (NaDT) photocatalyst (2.21 g, 1 mol %), achieving an isolated yield of 21.3 g (S)-γ-fluoroleucine (90%) with a 95% recovery rate of NaDT employing a COF Tp-DHBD membrane after 16 h of filtration.

The recycling of homogeneous photocatalysts in continuous flow systems can also be realized under a liquid–liquid biphasic environment, by combining solvents with distinct physiochemical properties (e.g., aqueous, organic, ionic liquids, and supercritical fluids) for the dissolution of photocatalyst and products.^80^ Hall et al. demonstrate continuous photocatalytic oxidation of α-terpinene and citronellol in a supercritical carbon dioxide (scCO_2_)-fluorous solvent system, achieving enhanced catalytic performance and efficient cycling of fluorinated photocatalyst after pressure releasing (Figure 3d).^89^ The scCO_2_ is an inert solvent with nonflammable and nontoxic properties, thus avoiding the consumption of photogenerated singlet oxygen (^1^O_2_). Additionally, the relatively long lifetime of ^1^O_2_ in scCO_2_ facilitates faster reaction kinetics due to an enhanced mass transfer in the single phase.^104^ Fluorinated molecular catalysts are required owing to their excellent solubility in scCO_2_, thus increasing the requirement to efficiently recycle these costly materials. In order to maximize the solubility of the fluorinated photocatalyst in scCO_2_ and the fluorous solvent (HFE-7500) and minimize its solubility in product, the 5,10,15,20-tetrakis­(pentafluorophenyl) porphyrin photocatalyst (TPFPP) is functionalized with “*fluorous ponytails*” (−(CF_2_)_7_CF_3_) to yield an F8 derivative. During the reaction, scCO_2_ is completely miscible with oxygen and forms a homogeneous supercritical monophase with the photocatalyst, organic substrates, and fluorous solvent, which improves the contact between oxygen, substrate, and catalyst molecules to enhance the reaction efficiency under 18 MPa pressure. The catalyst loading (65 mg) in this continuous-flow system is 20-fold less than the batch reactor, achieving >99% conversion of α-terpinene at the optimal pressure, though a gradual decrease in citronellol conversion is observed during 20 h of operation. The system can be operated continuously for 20 h to produce 240 mL of target product, corresponding to a turnover number (TON) of 27,000. After reaction, the mixture was depressurized for separation. The use of a thick sapphire tube reactor to resist the high pressure and the loss of fluorous solvent during depressurization may hinder its application at practical scales.

The biphasic separation of photocatalysts and products can be also realized by tuning the hydrophobicity of the catalyst in a water–oil system. Shao et al. report the employment of a superhydrophobic COF photocatalyst for continuous H_2_O_2_ synthesis from oxygen reduction in a water-α,α,α-trifluorotoluene system.^90^ The superhydrophobic PF-BTTA-COF photocatalyst is realized by introducing perfluoroalkyl chains using a 1H,1H-undecafluorohexylamine (UFHA) precursor reacting with the unreacted carbonyl sites of the synthesized BTTA-COF via condensation, which is synthesized through a solvothermal [4 + 3] condensation between 5,5′-(benzo­[c]­[1,2,5]­thiadiazole-4,7-diyl)­diisophthalaldehyde (BTDIPA) and 2,4,6-tris­(4-aminophenyl)-1,3,5-triazine (TAPT, Figure 3e). During the reaction, the aqueous and oil phases are pumped into a T-mixer, forming a segmented flow within the tubular channel. The generated H_2_O_2_ will migrate across the oil–water interface and enrich the adjacent aqueous segments. The collected liquid is spontaneously separated due to immiscibility, forming an upper aqueous H_2_O_2_ solution as the product and a lower catalyst-rich oil phase for reuse. By adjusting the flow rates, pH value, and concentration of the sacrificial agent (i.e., benzyl alcohol), a H_2_O_2_ production rate up to 968 μmol·h^–1^ with a maximum H_2_O_2_ concentration up to 38.1 mM can be achieved. This system shows potential for applications once the scaled synthesis of the superhydrophobic COF photocatalyst can be realized and the final concentration of H_2_O_2_ could be further geared up.

### 3.1 Immobilization of Photocatalysts

The packed-bed system offers the simplest solution for the fixation of photocatalysts. Ye et al. report the photocatalytic halogenation of hydrocarbons in a classic fixed-bed photoreactor by packing a quartz tube with thoroughly ground TiO_2_, inorganic halogen source (NaX, FeX_2_), silica gel, and sea sand (Figure 4a).^105^ It is proposed that FeX_2_ promotes the reduction of molecular oxygen and consumption of photogenerated reactive oxygen species (ROS) via the formation of FeX_3_, which participates in the halogenation of C–H bonds under irradiation and reduces back to FeX_2_ to complete the cycle. The NaX supplements the consumed halogen, thus avoiding the use of toxic elemental and organic halogen sources. A productivity of 1.2 mmol·g^–1^·h^–1^ with a high selectivity (>93%) to benzyl bromide from photocatalytic bromination of toluene is realized in a continuous flow system under 1 bar air, RT, 365 nm LED (75 mW·cm^2^), with a packing of 70 mg TiO_2_, 2.1 mmol FeBr_2_, 4.5 mmol NaBr, and 7 g silica gel. However, the simple and cost-effective packed-bed design may experience gradual leaching of expensive photocatalysts. In addition, severe light shielding and limited mass transfer within the central region of the packed bed reactor can potentially induce unwanted side reactions.

**Figure 4:**
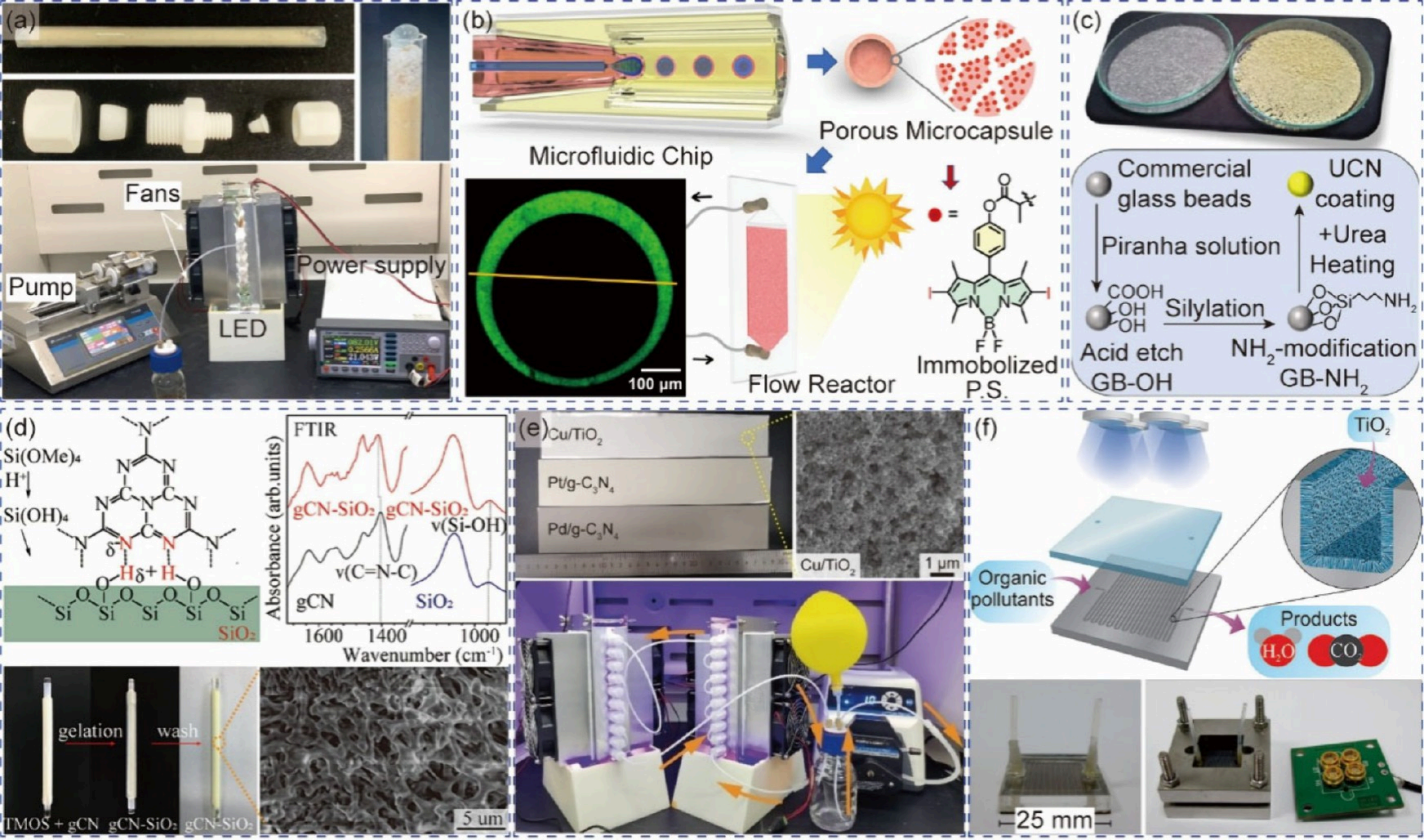
Immobilization of photocatalysts. (a) The assembled fixed-bed photocatalytic reaction system with TiO_2_, FeBr_2_, and NaBr for C–H bond halogenation. Reprinted with permission from ref 105. Copyright 2023, Wiley-VCH.^105^ (b) Fabrication of porous microcapsules with embedded MA-2IBDP photocatalyst. Reprinted with permission from ref 106. Copyright 2021, American Chemical Society.^106^ (c) Anchoring carbon nitride photocatalysts onto glass substrates through polymerization. Reprinted with permission from ref 107. Copyright 2020, Springer Nature.^107^ (d) Anchoring carbon nitride photocatalysts onto glass substrates via hydrogen bonds.Reprinted with permission from ref 108. Copyright 2023, Elsevier.^108^ (e) Photocatalyst membranes and the assembled tubular flow system. Reprinted with permission from ref 109. Copyright 2023, Wiley-VCH.^109^ (f) In-situ growth TiO_2_-based microreactor. Reprinted with permission from ref 110. Copyright 2013, American Chemical Society.^110^

The immobilization of photocatalyst can also be realized by confining it within a matrix for flow photosynthesis. Yang et al. report the fabrication of porous microcapsules with embedded methacrylate functionalized diiodo-Bodipy (MA-2IBDP) photocatalyst (Figure 4b).^106^ The wet-chemical derived MA-2IBDP can be copolymerized with the glycidyl methacrylate monomer and the trimethylolpropane triacrylate cross-linker, with 2-hydroxy-2-methylpropiophenone as the photoinitiator and 1-undecanol as a phase separation agent in a poly­(vinyl alcohol) (PVA) aqueous solution. The solution is injected into a designed dual-coaxial microfluidic chip using a syringe pump to produce monodisperse double-emulsion microdroplets, which was cured by LED for polymerization and washed with water and ethanol to remove PVA and the phase separation agent. Laser confocal microscopic imaging reveals a successful immobilization of MA-2IBDP on the surface of the microcapsule, forming a homogeneous shell with a thickness of ∼20–30 nm. A flat plate flow reactor filled with the porous microcapsules displays a 10-time enhanced rate constant for juglone synthesis (0.896 s^–1^) in comparison to the homogeneous system employing MA-2IBDP, and can also be employed for the aza-Henry reaction, Alder-ene reaction, and oxidation of thiols with satisfactory conversion rates (>95%). The decent catalytic performance is associated with an optimized light absorption, enhanced mass transfer, and prolonged lifetime of triplet excited-states. The performance under practical concentration of reactants and volume scale deserves further investigations for applications.

The anchoring of heterogeneous photocatalysts on transparent supports (e.g., glass beads, and fibers) is an alternative solution for immobilization. Yang et al. have grown graphitic carbon nitride (gCN) photocatalysts onto glass substrates through an *in situ* thermal polymerization method with 3-aminopropyltriethoxysilane (APTES) as a binder (Figure 4c).^107^ The cleaned and etched glass beads by Piranha solution are functionalized with amine groups via a silylation reaction using APTES, which are mixed with urea and heated to bond with the gCN via deamination. The loading of gCN photocatalyst is ∼1 wt %, forming a homogeneous layer with a flake-like structure and a thickness of ∼5–10 nm that fully covers the glass substrate. The photocatalytic [2 + 2] dimerization of α-asarone (0.167 M, 60 mL) in a continuous-flow mode is realized by packing the photocatalyst-coated glass beads (8 g) in a flow photoreactor. A high conversion (89%) is achieved after 48 h of white LED irradiation (0.1 W·cm^–2^), yielding 1.6 g of magnosalin. The system can be operated for five cycles without obvious decrease in performance, attributed to the strong binding between the glass beads and the gCN photocatalyst, thus offering a solution for continuous production of pharmaceutical compounds. Liu et al. propose the anchoring of gCN on SiO_2_ via hydrogen bonds through a sol–gel process, providing shaped photocatalyst with porous structure for enhanced light absorption and mass transfer (Figure 4d).^108^ This is realized by injecting the colloidal sol that contains as-synthesized gCN powders, poly­(ethylene oxide) (PEO), sodium dodecyl sulfate (SDS), tetramethoxysilane (TMOS) and propylene oxide (PO) into a PTFE mold that contains a centered quartz glass rod, which undergoes a sol–gel transition at 45 °C for 36 h and yields a glass rod coated with gCN-SiO_2_ after removing the mold. During the gelation process, long-chain porous silica is formed upon dehydrative condensation of TMOS, and the triazine units of gCN are spontaneously anchored to the as-generated silica skeleton via multiple hydrogen bonds according to infrared spectroscopy. The gCN distributes homogeneously throughout the porous silica without completely blocking the pore, as the specific surface area of silica only slightly decreased after loading gCN. This strong interaction enables the gCN photocatalysts to remain stable under flushing without detachment. The glass rods with gCN-SiO_2_ are inserted into quartz glass tubes for the photocatalytic reductive coupling of nitrobenzene, achieving selective synthesis of azoxybenzenes at a gram-scale that outperforms suspension flow using gCN and packed-bed flow using gCN coated on glass beads under identical reactions conditions. Interestingly, kinetic analysis reveals that the gCN-SiO_2_ flow system prolongs the lifetime of nitrosobenzene and hydroxylamine intermediates owing to the well dispersed active sites within the porous skeleton of the gCN-SiO_2_, resulting in a delayed coupling process that avoids unwanted Wallach rearrangement of azoxybenzene under excessive irradiation. The mechanical integrity of gCN-SiO_2_ is well maintained after five consecutive cycles under a high flow rate according to XPS analysis on the N 1s/Si 2p peak ratio, confirming the stability of the hydrogen-bonded gCN on SiO_2_ scaffold.

The construction of a porous film with only a photocatalyst could further benefit light absorption and mass transfer of reactants. This is especially crucial for the efficient utilization of some precious photocatalyst materials. **Li** et al. present a generalizable method for the preparation of porous photocatalyst membrane coated on aluminum foil for direct use in a modular tubular flow system (Figure 4e).^109^ The film is simply realized by blade-coating of a homogeneous slurry that contains the photocatalyst (i.e., TiO_2_, gCN, and CdS), binder (polyvinylidene fluoride, PVDF), and pore-forming agent (*N*-methyl-2-pyrrolidone, NMP) in a specific ratio onto the aluminum foil. A mild drying at 80 °C for 12 h is sufficient for the removal of NMP, resulting in a porous photocatalyst membrane with a thickness of ∼5–10 μm according to scanning electron microscopy (SEM), which is sufficient for the complete absorption of incident light in comparison with the powder samples. This corresponds to a catalyst loading of ∼3–4 g·m^–2^. Remarkably, the specific surface areas for the photocatalyst membranes are very close to those of the original photocatalyst powders (i.e., 49.0 m^2^·g^–1^ for the Cu/TiO_2_ membrane and 50.7 m^2^·g^–1^ for the Cu/TiO_2_ powders), thus maximizing the number of accessible active sites and ensuring an optimum mass transfer during photocatalytic reactions. The film is then wrapped on glass rods and inserted into tubes for photocatalytic dehalogenative coupling of benzyl bromide, dehydrogenative coupling of benzylamine, and hydrogenation of benzaldehyde, which can be connected in series or parallel for scaling-up under LED or solar irradiation. Interestingly, these photocatalytic reactions all undergo zeroth-order reaction kinetics, completing the reaction ∼6 times faster than the batch system under otherwise identical reaction conditions (i.e., light intensity, reaction volume, concentration, and loading of photocatalyst). This is associated to full utilization of active sites and light absorption, which endorse the system a satisfactory yield and QE even under high concentration of reactants (500 mM) and large reaction volume (200 mL). The membranes also show decent mechanical stability after ∼200 h of continuous operation, yet leaching of the metal cocatalyst supported on the semiconductor photocatalyst is observed (i.e., Cu, Pt, and Pd). The leaching of metal mainly originates from a weak interaction of metal with the semiconductor support, which can be minimized by constructing stronger bonds between metals and supports,^111^ encapsulating metal nanoparticles within protective layers,^112^ and embedding metals in porous matrices.^113^ A reduced level of employment of solvents and mechanical force on the immobilized heterogeneous photocatalysts in flow may reduce the leaching rate. Additionally, the flow system also facilitates online monitoring of leached metal in solution, thus benefiting the optimization of catalyst design and operational conditions. Nevertheless, the simple preparation of photocatalyst membranes and construction of modular tubular flow systems have been successfully adopted in the gram-scale photosynthesis of phenol and juglone using MOFs photocatalyst,^114^ high purity pinacols and amides in a tandem flow system using CdS@PCN-Co and Pd/PCN-Zn photocatalysts,^115^ and photothermal CO_2_ cyclization using COFs photocatalyst.^116^

The immobilization of a heterogeneous photocatalyst can be also realized in a microchannel reactor. Krivec et al. report the *in situ* growth of TiO_2_-based photocatalyst within the channels of a microfluidic device for photocatalytic degradation of organic pollutants (Figure 4f).^110^ The microchannel is mechanically engraved onto a titanium foil substrate, and a double-layered TiO_2_ anatase film is immobilized on the inner walls via anodization and subsequent hydrothermal treatment. This results in the development of an ∼10 μm TiO_2_ layer that consists of titania nanotubes and anatase NPs, which is firmly attached to the titanium substrate. The resulting square-shaped catalytic module with a size of ∼25 mm is housed in a stainless-steel case with four UV-LEDs (∼1.2 mW·cm^–2^) for photocatalytic degradation of caffeine. The system displays an initial decomposition rate of 1.06 mmol·L^–1^·h^–1^ and an optimum QE of ∼0.4% for the decomposition of 25 mg·L^–1^ at a flow rate of 40 μL·min^–1^. The catalytic performance of the microreactor can be maintained at ∼60% of its initial catalytic activity after an operation over 6 months (3,600 cycles), demonstrating a reasonable stability. The degradation of performance may be attributed to the progressive dissolution and structural reconstruction of the TiO_2_ nanoparticles under prolonged exposure to the reaction medium and the byproducts, resulting in a reduction of active sites. Therefore, the recovery and scaling-up of the system are crucial for wider applications.

Recently, the rapid development of 3D printing techniques with improved quality and reduced cost boosts the construction of very complicated structures from macro- to gigantic- scale.^117,118^ This is especially of great interest for the build-up of microchannel reactors for photocatalysis systems using transparent resins.^119^ Zhang et al. fabricate a fluorescent fluidic photochemical microreactor (FFPM) using a stereolithography based 3D printer,^120^ which consists of both reaction channels for photocatalysis and light emitting channels filled with fluorescent liquid dyes (Figure 5a). Stereolithography is a laser-based point-by-point photopolymerization 3D printing method that cross-link monomers and oligomers to create layer-by-layer structures with submicron accuracy.^121^ The fluorescent medium captures photons that cannot be absorbed by the photocatalyst and converts them through up- and down- conversion to match the bandgap of the photocatalyst. This design allows for simple replacement of fluorescent dye molecules with characteristic absorption and emission spectra to suit specific photocatalysts and reactions. Additionally, the catalytic performance can be tuned by optimizing both reaction and light emitting channels, which manipulate the distribution of illuminance and the concentration field of reactants. Representatively, the cycloaddition of 9,10-diphenylanthracene (DPA) with molecular oxygen using methylene blue as the photosensitizer (MB, λ_max_ = 654 nm) is employed as a model reaction, with fluorescent perylene bisimide-based dye (Lumogen F red 305, LR305) as a down-conversion luminophore. The LR305 dye absorbs visible light in the range of 400–620 nm and emits light peaked at ∼630 nm, thus allowing the excitation of MB to drive the reaction using a 440 nm LED. The FFPM shows a decent conversion of 70% at a retention time of 86 s using 400 ppm of LR305 as the fluorescent liquid, which is 3.5 times higher under direct irradiation. The dehydrogenative coupling of *p*-thiocresol can be also realized by using EY as the photosensitizer and 400 ppm LR305 as the fluorescent liquid.

**Figure 5:**
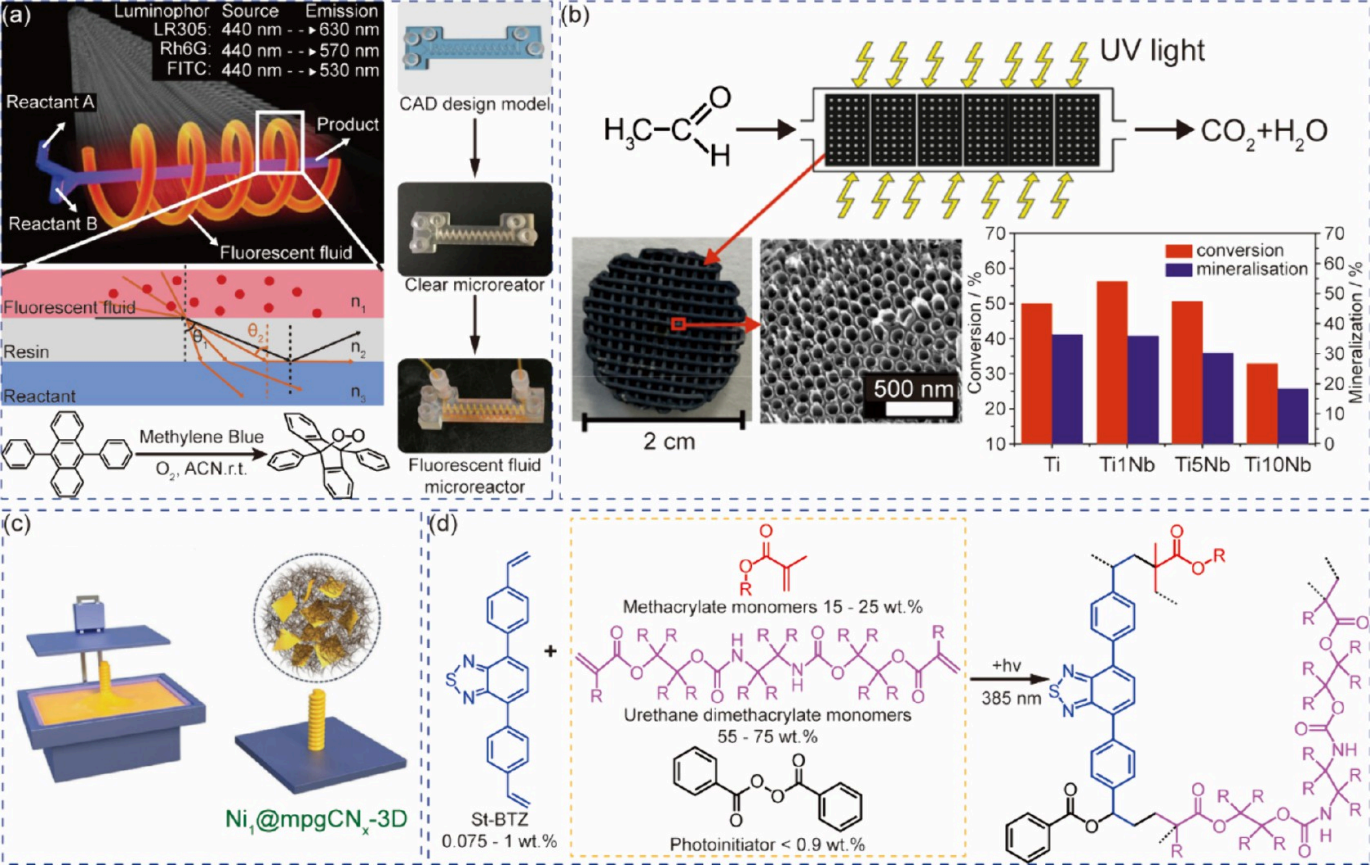
3D printing for photocatalysis in flow. (a) Fluorescence-fluidic photochemical microreactor prepared by stereolithography. Reprinted with permission from ref 120. Copyright 2019, Wiley-VCH.^120^ (b) 3D Ti–Nb grids with TiO_2_ nanotubes (TNTs) surface prepared by direct ink writing combined with wireless anodization. Reprinted with permission from ref 122. Copyright 2023, American Chemical Society.^122^ (c) A structured single-atom catalyst prepared by masked stereolithography. Reprinted with permission from ref 124. Copyright 2024, Wiley-VCH.^124^ (d) A monolithic structured photocatalyst coupled with photocatalytic monomer and photosensitive resin by digital light processing. Reprinted with permission from ref 125. Copyright 2021, Elsevier.^125^

Alternatively, the as-printed metallic alloy 3D meshes can be converted to photocatalyst materials for use in flow reactors. Sopha et al. report the use of direct ink writing (DIW) additive manufacturing technology for the preparation of 3D Ti–Nb alloy meshes from Ti–Nb powder mixtures, which are electrochemically anodized to form a TiO_2_ nanotube-covered surface for photocatalytic degradation of acetaldehyde in a flow-through gas phase reactor (Figure 5b).^122^ The composition of the alloy can be simply adjusted by controlling the ratio of Ti and Nb powders, thus providing a tool to control the doping level of Nb in TiO_2_ nanotubes and improving the compressive strength of the mesh. The 3D grid Ti–Nb structures (20 × 8 mm) present a uniform filament spacing of ∼857 μm and a porosity of 68%, providing channels for the diffusion of reactants. The surface of anodized and annealed 3D meshes displays a homogeneous distribution of arrayed TiO_2_ nanotubes with diameters of ∼80 nm and thickness of ∼1.5–7 μm. The flow reactor with six pieces of anodized 3D grids in a quartz tube is employed for the decomposition of acetaldehyde (5 ppm) under UV irradiation, achieving an optimum mineralization efficiency of ∼86% at a low flow rate (0.5 L·min^–1^).

It is also possible to deposit heterogeneous photocatalysts on the inner walls of 3D printed microreactors,^123^ though their homogeneity and adhesion need to be considered. Luo et al. show the preparation of structured single-atom Ni immobilized mesoporous graphic carbon nitride (Ni_1_@mpgCN_*x*_-3D) via photopolymerization without any intermediate washcoating step for photocatalytic oxidation of benzyl alcohol (40 mM, Figure 5c).^124^ A helically twisted structure imitating a Kenics static mixer (twist angle of 45°) is printed using a “ink” that contains the presynthesized Ni_1_@mpgCN_*x*_ powders and commercially available photosensitive resin in a mass ratio of 1:100. The structured photocatalyst embedded in a tubular continuous-flow photocatalytic reactor shows a production rate of 20 mg_prod_ mg_cat_^–1^·h^–1^ for benzaldehyde under 465 nm LED irradiation in acetonitrile at RT with a flow rate of 3 mL h^–1^, which is better than the traditional packed-bed reactor (14 mg_prod_ mg_cat_^–1^·h^–1^) and the mpgCN_*x*_-3D (18 mg_prod_ mg_cat_^–1^·h^–1^). This enhancement is attributed to the spiral architecture of the structured photocatalyst, which is optimized by using CFD simulations coupled with radiation transport models. The helical design promotes fluid recirculation and turbulent mixing, leading to improved mass transfer and enhanced light distribution within the reactor. The marginally enhanced performance may be associated with the wrapping of active sites by the polymer, and a slightly reduced catalytic performance during the stability test may arise from the dissolution of photocatalyst in solvent.

Zhakeyev et al. propose to develop a print ink recipe that consists of the photocatalyst monomer, the skeleton monomers, and the photoinitiator for direct anchoring of the catalyst during polymerization, thus avoiding the blocking of active sites for catalytic reactions.^125^ Representatively, the vinyl groups of the photocatalytic monomer [4,7-di­(4-vinyl)­phenylbenzo-2,1,3-thiadiazole. (St-BTZ)] can copolymerize with the vinyl groups of commercially available methacrylate and urethane dimethacrylate monomers upon 385 nm irradiation with benzoyl peroxide as the photoinitiator (Figure 5d). The optimal loading of St-BTZ is determined to be 0.19 wt % according to the intensity of C=C stretching vibrational peaks (1620–1650 cm^–1^) by infrared spectroscopy, which provides a decent light absorption up to ∼475 nm. The shaped photocatalyst with four cylindrical monolithic designs with variable channels and periodic void spaces is designed and fabricated employing a digital light processing (DLP) 3D printer, achieving a printing accuracy exceeding 80%. All fabricated monolith designs can be used for photocatalytic conversion of 2-furoic acid to γ-lactone (5-hydroxy-2­(5H)-furanone) through singlet oxygen mediated oxidation in continuous flow reactor, where the simplest design (D2) of monolith with parallel channels shows an optimum STY at a high flow rate (2 mL·min^–1^). This is possibly due to a minimized maldistribution of the flowing gas and liquid phases rather than mass transfer at the monolith surface.

## 4 Photon Economy: Sunlight Harvesting

Although artificial light sources (e.g., Xenon lamps and LEDs) provide a reliable energy input for continuous operation of photocatalytic reactions, it is always desired to drive the reactions by utilizing sunlight as the ultimate sustainable energy source. The transition to efficient solar-driven flow systems hinges on three interconnected parameters: (1) designed reactors for optimized light penetration; (2) engineered photocatalyst and photosensitizer for solar light absorption; and (3) simplified recycling and regeneration of photocatalyst. This is especially critical for photocatalytic water splitting, CO_2_ reduction, N_2_ fixation, and pollutant degradation, which are very sensitive to the cost of electric energy. Since the selection of photocatalysts for specific redox reactions must match the thermodynamic redox potentials of reactant substrates, only a certain portion of photons in the solar emission spectrum can be utilized. Current research is focused on the design and scaling-up of flow systems for efficient sunlight absorption using established photocatalyst materials and maximizing utilization of the full solar output via incorporation of up- and down- conversion materials. For synthetic chemistry, the selectivity to target products may also be sensitive to wavelength,^126^ thus requirements for light filtration may also need to be considered. Furthermore, the efficiency of sunlight-driven photocatalysis could also be constrained by the inherent limitations of solar radiation, including variable light intensity and high IR content, limiting practical implementation of photocatalysis for nonstop operations.^127,128^ Our previous work shows that the bulk system is simply inappropriate for efficient utilization of solar energy in the synthesis of value-added azoxy aromatics, due to the limited penetration depth of sunlight and unwanted side reactions under dark conditions.^126^

Flat plate flow reactors embedded with established semiconductor photocatalyst membranes is the most widely applied solution for solar-driven hydrogen production, CO_2_ reduction, and pollutant degradation, owing to the simple fabrication for scaling-up and large illumination geometric area for light absorption.^129–131^ Zhao et al. propose the “Hydrogen Farm Project (HFP)” that consists of solar energy capturing and hydrogen production subsystems integrated by a shuttle ion loop.^132^ This system mimics natural photosynthesis, employing BiVO_4_ photocatalysts with precisely tuned facets for water oxidation reactions coupled with the reduction of Fe^3+^ to Fe^2+^, which is injected into an electrolyzer to oxidize back to Fe^3+^ accompanied by hydrogen production on the Pt cathode. The overall reaction is solar-driven water splitting. The decahedron BiVO_4_ photocatalyst shows a high AQE (up to 71%) for oxygen evolution from water oxidation within its absorption spectrum (<500 nm), with the addition of 4.0 mM Fe­(NO_3_)_3_ as electron scavenger (Figure 6a). The shaped BiVO_4_ photocatalyst powders (12 g) are directly brushed onto a commercial biaxially oriented polypropylene (BOPP) film with an acrylic adhesive layer (1.0 × 1.0 m^2^), yielding a photocatalyst film to assemble a 1 m^2^ flat plate flow reactor for water oxidation under sunlight conditions in an aqueous Fe­(NO_3_)_3_ solution (32 mM). The hydrogen evolution can be operated in either a photoelectrochemical or an electrochemical cell in a 1 M H_2_SO_4_ electrolyte, showing an overall solar-to-chemical efficiency over 1.9% and a solar-to-hydrogen (STH) efficiency exceeding 1.8%. The concentration of Fe^2+^ generated matches well with the concentration of consumed Fe^3+^, suggesting that Fe^2+^ can be obtained quantitatively under practical sunlight conditions. A reaction equilibrium of Fe^2+^/Fe^3+^ seems to be reached during the reaction, implying that photogenerated Fe^2+^ should be used immediately in the electrochemical hydrogen evolution to achieve an optimized efficiency.

**Figure 6:**
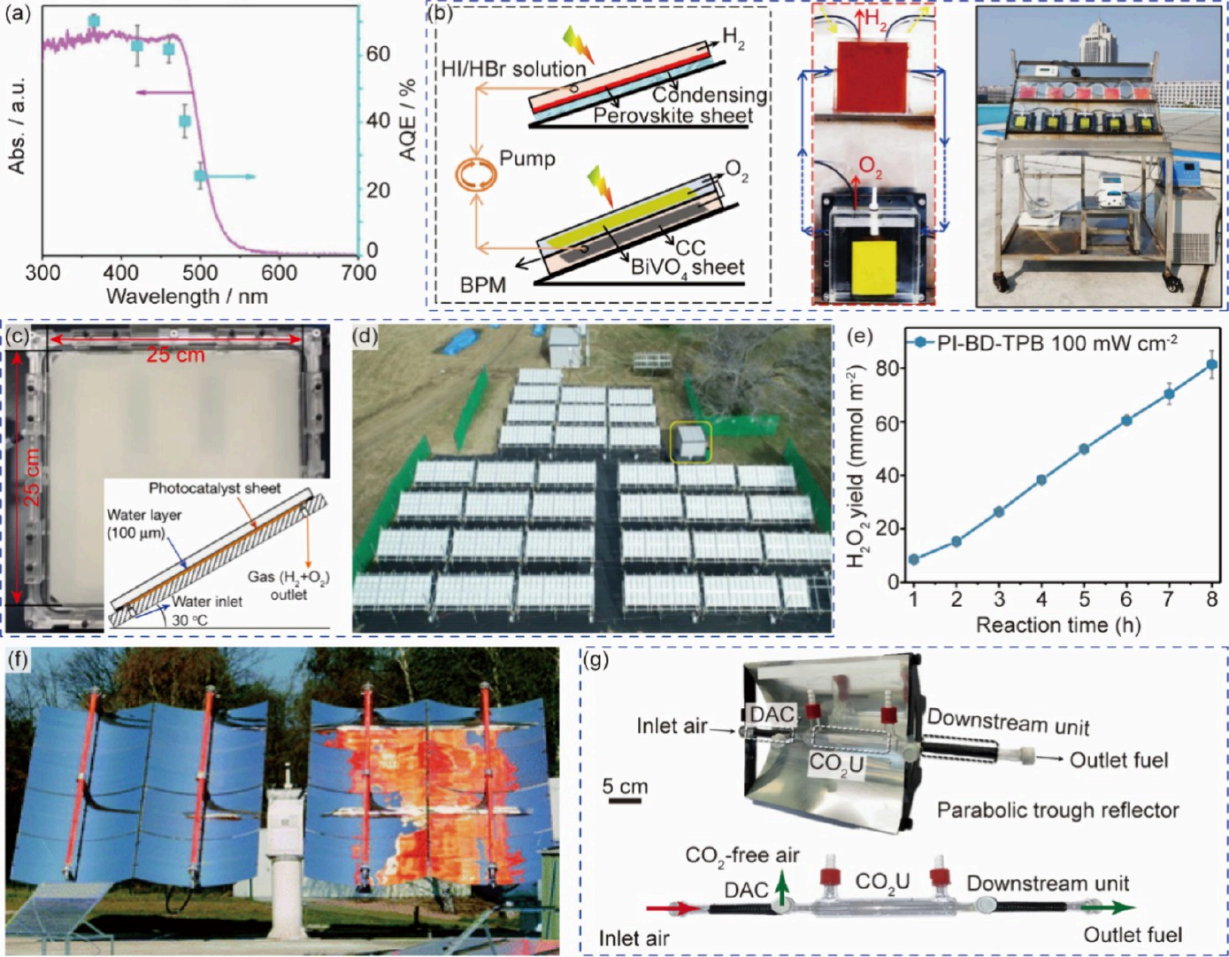
Reactors for solar-driven photocatalysis. (a) Absorption spectra and AQE of the shaped BiVO_4_ photocatalyst for photocatalytic water oxidation coupled with the reduction of Fe^3+^. Reprinted with permission from ref 132. Copyright 2020, Wiley-VCH.^132^ (b) Scheme and image of a tandem unit for separated water splitting, and a 5-module system for solar-driven water splitting system. Reprinted with permission from ref 133. Copyright 2025, Springer Nature.^133^ (c) and (d) Image and structure of a panel reactor unit (625 cm^2^) for the 100 m^2^ solar hydrogen production system. Reprinted with permission from ref 134. Copyright 2021, Springer Nature.^134^ (e) Practical performance of an aerogel photocatalyst membrane for H_2_O_2_ production. Reprinted with permission from ref 135. Copyright 2024, Springer Nature.^135^ (f) PROPHIS reactor for photo-oxygenation of citronellol. Reprinted with permission from ref 136. Copyright 2005, Springer Nature BV.^136^ (g) The designed flow reactor with direct air carbon capture (DAC) and CO_2_ utilization (CO_2_U) units placed in a parabolic solar concentrator. Reprinted with permission from ref 137. Copyright 2025, Springer Nature.^137^

Alternatively, separated solar-driven hydrogen and oxygen evolution from water can be realized using the flat plate flow reactors by coupling a hydrogen evolution cell and an oxygen evolution cell, as presented by Fu and coauthors.^133^ The photocatalytic H_2_ evolution and the O_2_ evolution cells are separated and mediated by the I_3_^–^/ I^–^ redox shuttle (Figure 6b). The mixed halide perovskites FAPbBr_3‑x_I_*x*_ (FPBI, FA=CH­(NH_2_)_2_^+^) loaded with molybdenum selenide (MoSe_2_) is employed as the photocatalyst for H_2_ production, accompanied with the oxidation of I^–^ to I_3_^–^. Meanwhile, the NiFe-layered double hydroxide modified BiVO_4_ (NiFe-LDH/BiVO_4_) film grown on a fluorine-doped tin oxide (FTO) glass is used as the photocatalyst for water oxidation with the linked carbon cloth (CC) for the reduction of I_3_^–^. Since the oxygen evolution cell does not contain the redox mediators, the side reactions caused by the redox couple are avoided. The flat panel flow reactor modules consist of 10 × 10 cm^2^ perovskite-based photocatalyst immobilized on an acrylic plate and NiFe-LDH/BiVO_4_//CC films (5.5 × 7 cm^2^) are connected and assembled in series (5 modules) for outdoor exemplification, resulting in stoichiometric H_2_ and O_2_ evolution rates of 105 mL·h^–1^ and 49 mL·h^–1^, corresponding to an averaged STH efficiency of 1.21% during a week-long test under natural sunlight. The integrated flow system facilitates the removal of generated I_3_^–^ from the hydrogen evolution cell to the oxygen evolution cell, thus avoiding the build-up of I_3_^–^ that blocks the light absorption of the photocatalyst. This design prevents the use of additional reductant (e.g., H_3_PO_2_), though a slightly reduced hydrogen evolution rate is still observed at longer operation times due to the increased concentration of I_3_^–^. Additionally, the authors also observe the gradual leaching of the immobilized photocatalyst and the incomplete usage of I_3_^–^ in the oxygen evolution reaction.

Direct sunlight-driven water splitting in flat plate reactors at a practical scale has also been reported by Nishiyama and coauthors. The flat plate module has an optical window of 625 cm^2^, which contains a frosted glass sheet with spray-coated photocatalyst nanoparticles (modified SrTiO_3_:Al) with a thickness of 4–10 μm (Figure 6c).^134^ To provide more accessible active sites for water splitting, the photocatalyst layer exhibits a mesoporous structure by immobilizing the photocatalysts on the silica nanoparticles. An ultraviolet-transparent glass window is placed above the photocatalyst sheet to seal the module, creating a space of 0.1 mm for channels for water and gas flow. This minimizes the loading of water and prevents accumulation and ignition of the evolved oxyhydrogen gas (hydrogen–oxygen mixture). The module is tilted at a 30° angle to facilitate the liberation of gases. A 100 m^2^ flow system has been constructed by arraying 1,600 of such modules and an in-line gas separation facility with commercial polyimide hollow-fiber membrane modules (Figure 6d). The peak H_2_ production rate reaches 3.6–3.7 L min^–1^ with a maximum STH efficiency of 0.76% (recorded between Sep. 22nd to Dec. 21st 2020), with a H_2_ purity of >94% and a recovery rate of 73%. A seasonal degradation of the STH efficiency to ∼0.3% in mid-December is observed, attributed to the reduced UV fraction in sunlight and physical detachment of the catalyst layer caused by repeated freezing-thawing cycles. Additionally, the deactivation of the photocatalyst during long-term operation (1600 h) caused by the relocation of impregnated chromium components onto Rh sites of the SrTiO_3_:Al is also observed.^138^ The authors also point out the negative net energy balance of this system as the energy consumed by the diaphragm pump for gas-separation (6.1 MJ) exceeds the energy of the produced hydrogen (5.0 MJ).

The flat plate reactor can also be employed for synthetic chemistry after appropriate modification, i.e., flow control, temperature control, light filtration, and anticorrosion protection. Chi et al. report the solar-driven synthesis of H_2_O_2_ from oxygen reduction by embedding a polyimide aerogel photocatalyst (PI-BD-TPB) membrane into a flat plate reactor.^135^ The cross-linked donor–acceptor structured polymer consists of photoreductive carbonyl groups, enabling the activation of molecular oxygen to produce H_2_O_2_ in the absence of sacrificial agents (i.e., alcohols) through its redox cycle. The PI-BD-TPB photocatalyst also displays durable performance up to 144 h with a decent solar-to-chemical conversion efficiency (SCC) of 0.92% under a saturated O_2_ atmosphere. The macroscopic PI-BD-TPB aerogel membrane exhibits a H_2_O_2_ yield of 88.6 mmol·m^–2^ in 8 h of irradiation under a solar simulator (100 mW·cm^–2^, Figure 6e), and can be scaled-up to a 0.5 m^2^ system with a H_2_O_2_ yield of 34.3 mmol·m^–2^. Yang et al. report the utilization of a ZnIn_2_S_4_ photocatalyst with atomically dispersed Pt cocatalyst for solar-driven dehydrogenative homocoupling of benzylamine in a 75 × 75 cm^2^ flat plate flow reactor.^139^ The photocatalyst film was prepared by a simple drop coating technique on nonwoven fiber according to a previous work.^140^ A total volume of 125 mL of imine product with a purity of 97 wt % can be produced after 2 weeks of outdoor irradiation, though some key parameters are not disclosed for comparison and evaluation (e.g., catalyst loading, temperature control, regulations on gas evolution). Additionally, the efficiency for solar light utilization can be improved by simply introducing a reflective layer at the rear side of the reactor (i.e., silver coating or a mirror), thus redirecting the transmitted light back into the reaction zone.^141^ An example is the previously discussed modular tubular system by Li et al., where a reflective aluminum plate is mounted on the back-side of the module to reflect back unabsorbed photons.^109^ Representatively, the solar-driven homocoupling of benzylamine (40 mM, 50 mL) employing a Pt/g-C_3_N_4_ membrane (80 mg) completes within 18 h of irradiation, displaying a three-time enhanced yield of product compared to a traditional batch reactor under comparable reaction conditions. Since the power consumption of the peristaltic pump is only 14 W, a complete solar-driven synthetic system can be realized by coupling the modular tubular flow system with a solar panel. Nevertheless, the filtration of solar irradiation may need to be considered for specific reactions to avoid reduction of product selectivity and degradation of photocatalyst. We show the necessity of applying a transparent polymer cover to remove the UV portion of sunlight in the photocatalytic reductive coupling of nitroaromatics, which improves the selectivity in the synthesis of azo- and azoxy- compounds (>95%) by avoiding the formation of hydroxylated azobenzene *via* the Wallach rearrangement.^108^

The irradiance of the solar radiation varies depending on the wavelength, but is in the range of 0.5–1.2 W·m^–2^·nm^–1^ at sea level on a clear day.^142^ It is therefore necessary to concentrate the density of the photon flux to accelerate some reactions with inherently slow kinetics. The parabolic trough-facility for organic photochemical syntheses (PROPHIS) is developed to address these shortcomings (Figure 6f).^136^ The facility is located at the solar-chemical facility of the German Aerospace Center (DLR, 70 m above sea level), equipped with four silver-coated parabolic glass troughs as the solar concentrator (total area of 32 m^2^) for placing the photoreactors (35–120 L). This provides a geometric concentration factor equivalent to the output from 32 suns, according to the ratio of the collector aperture area to the absorber area. Oelgemöller et al. have employed the PROPHIS for photooxygenation of citronellol in an isopropanol solution under oxygen-rich conditions.^143^ Here rose bengal is employed as the photosensitizer owing to its decent light absorption (maximum at 555 nm). A full conversion (>95%) of citronellol (8.0 L, 43.9 mol, 6.8 kg) is realized within 3 h of solar irradiation, employing only 36 g of rose bengal and 72 L of isopropanol. This corresponds to a total receiving of 133.4 mol photons in the range of 500–600 nm. The regioisomeric products are formed in a ratio of ∼45:55, which is in good agreement with reported isolated yields from laboratory experiments using artificial light. It is believed that a better efficiency can be obtained by improving the supply of molecular oxygen inside the reactor. In addition, the synthesis of Juglone (5-hydroxy-1,4-naphthoquinone, 4) from 1,5-dihydroxynaphthalene is also achieved with a small parabolic trough collector equipped with holographic mirrors (20 × 100 cm^2^), which are designed to reduce warm-up effects caused by infrared radiation. Juglone with an isolated yield of 79% is achieved after an illumination period of ∼9.5 h, which is much faster when compared to the reaction performed without a solar concentrator (several days or weeks). However, the STY of the PROPHIS system should be considered for practical applications. Similarly, the compound parabolic concentrator (CPC) with an involute reflective surface around cylindrical reactor tubular reactors has been also developed for solar-driven photocatalysis.^144^ This cost-effective configuration is ideal for the capture of direct and diffuse UV-sunlight with low intensity with a concentration ratio close to one, which is often used for pollutant decomposition and oxidative conversion of chemicals employing wide bandgap photocatalysts (e.g., TiO_2_).^145–148^ A representative work by Spasiano et al. report the sunlight-driven selective oxidation of benzyl alcohol to benzaldehyde employing a pilot-scale system with 12 CPC units.^149^ The yield of benzaldehyde reaches 53.3% for the conversion of a 39 L benzyl alcohol solution (1.5 mM) using TiO_2_ as the photocatalyst and CuSO_4_ as the electron scavenger under deaerated conditions in 385 min of solar irradiation. However, the low concentration of the reactant and the required regeneration of Cu^2+^ limit its applications.

Very recently, the solar concentrator has been successfully embedded in solar-driven CO_2_ capture and conversion (Figure 6g).^137^ The system designed by Kar et al. consists of a gas-phase direct air carbon capture and utilization (DACCU) flow reactor and a parabolic trough reflector. The DAC unit is packed with a solid silica-polyamine CO_2_ adsorber, which selectively captures CO_2_ from the air at RT by chemisorption, and releases the captured CO_2_ at 80–100 °C. This enables the capture of CO_2_ during the nighttime and desorption upon heating by the photothermal effect of the solar concentrator, which diffuses to the CO_2_U unit filled with alumina/silica–titania-cobalt bis­(terpyridine) (Al_2_O_3_ /SiO_2_|TiO_2_|CotpyP) hybrid material for solar-driven CO_2_-to-fuel synthesis. The system produces ∼200 μmol of H_2_ and CO in a ∼1:1 ratio per gram of TiO_2_ within an irradiation time of 20 h under 1 sun (AM 1.5G) with the presence of ethylene glycol (EG) as the reductant for the CO_2_ reduction. It is also possible to employ poly­(ethylene terephthalate) (PET) waste to produce EG for the production of syngas with formate and glycolaldehyde dimer as side products, achieving a total CO quantity of ∼1,250 μmol per gram TiO_2_ over 96 h under 3-sun illumination at RT.

It is still essential to gear up the intrinsic performance of the photocatalyst to couple with established reactors, thus achieving optimized efficiency under solar irradiation. This includes optimization of the reaction kinetics and the extension of light absorption. Barzegar et al. have employed an S-scheme gC_3_N_4_/TiO_2_ heterojunction photocatalyst for solar-driven decomposition of a methylene blue (MB)-rhodamine B (RhB) mixture in a suspension flow mode with a parabolic concentrator.^150^ The composite system displays a removal efficiency of >90% for both dyes (ppm level) upon exposure to solar irradiation for 100 min owing to an efficient charge separation between gC_3_N_4_ and TiO_2_, though the dye decolorization is a complicated and ambiguous reaction for evaluation. Zhou et al. develop a hydrophilic hydrogen-bonded organic framework (HOF) with one-dimensional micropore channels [1,3,6,8-tetrakis­(*p*-benzoic acid)­pyrene (HOF-H_4_TBAPy)] to shorten the charge transfer path from the bulk exciton coupling region to the catalyst surface.^151^ The 1D micropore HOF is simply synthesized by adjusting the precipitation conditions of amorphous H_4_TBAPy in the solvent. The narrowing of the peaks related to the benzoic acid group observed for the HOF implies a restricted rotation of the σ bonds by the ordered framework structure (Figure 7a), due to the interlaminar π–π stacking and in-plane hydrogen bonding according to X-ray photoelectron spectroscopy (XPS) and infrared analysis. Additionally, low electron dose cryoelectron microscopic imaging and N_2_ adsorption isotherms confirm the ordered 1D microporous structure with a lattice spacing of 2.06 nm, an averaged pore size of 1.5 nm, and a π–π stacking distance of 0.36 nm. The length of the HOF can be precisely controlled by tuning the degree of protonation during crystal growth, resulting in a gradual decrease in contact angle following shortening of the HOF-H_4_TBAPy. The platinized HOFs show a high evolution rate of H_2_ with ascorbic acid as the sacrificial agent at shorter lengths, and exponentially decrease following the increase of the length (Figure 7b). Interestingly, the opposite trend of the quantum yield of photoluminescence (PLQY) is observed. Here the length of the active adsorption region (L_act_) is a threshold that corresponds to the maximum doubled length of the 1D pore channel, implying that the photoactivity is strongly associated with the micropore-confined exciton domain and the accessible active sites for water and ascorbic acid from both ends of the channel (Figure 7c). Since the inner surface of the micropore in the inert adsorption region is insufficient to quench all excitons due to poor accessibility, the migration of generated excitons to the active adsorption region results in losses through radiative or nonradiative recombination pathways.

**Figure 7:**
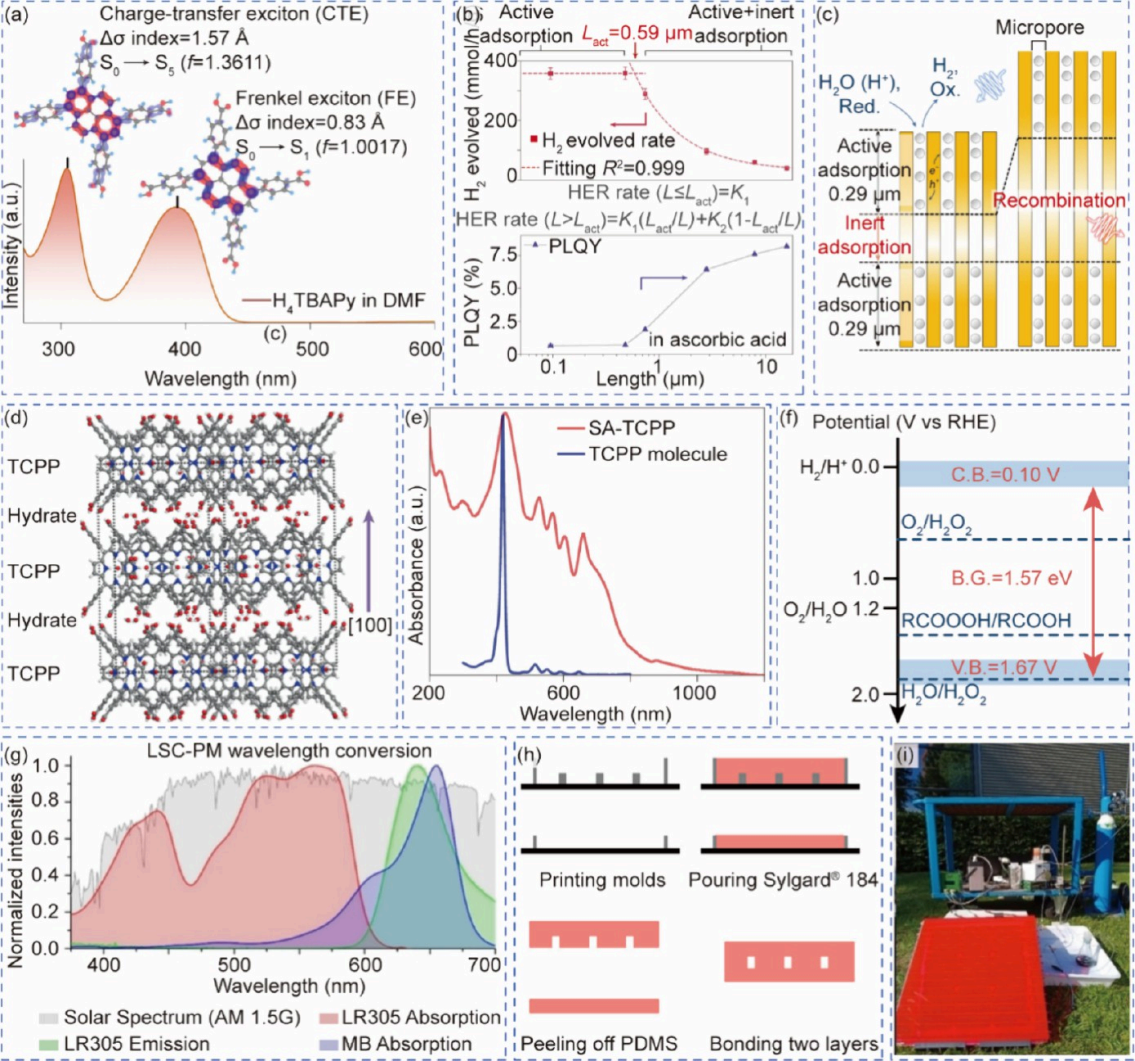
Materials for solar-driven photocatalysis. (a) ssNMR spectra and structure diagram of HOF-H_4_TBAPy and amorphous H_4_TBAPy powder. (b) H_2_ evolution rate and PLQY as a function of the mean length of the 1D microporous channel of the HOF-H_4_TBAPy. (c) Scheme of the HOF-H_4_TBAPy adsorbing water and hole scavenger. Reprinted with permission from ref 151. Copyright 2023, Springer Nature.^151^ (d–f) Crystal structure, light absorption, and band positions of the SA-TCPP supramolecular assembly for photocatalytic H_2_O_2_ production. Reprinted with permission from ref 152. Copyright 2023, Springer Nature.^152^ (g) Wavelength conversion scheme of the LR305/MB based LSC-PM. (h) Fabrication process of the LSC-PM. Reprinted with permission from ref 153. Copyright 2018, American Chemical Society.^153^ (i) Photographs of the off-grid solar-powered chemical mini-plant. Reprinted with permission from ref 154. Copyright 2021, Wiley-VCH.^154^

Zhang et al. report an extended light absorption by self-assembly of the tetrakis­(4-carboxyphenyl)­porphyrin molecule (TCPP).^152^ The wet chemical crystallization of the TCPP monomer is performed at 353 K for 3 days in an aqueous solution, resulting in the formation of self-assembled TCPP (SA-TAPP) nanosheet with exposed (014) and (032) planes that is stacked along the [100] direction (Figure 7d). The crystallization of porphyrin not only improves the charge transfer but also exhibits three types of micropores (∼0.8, 0.6, and 0.4 nm) that facilitate the diffusion of H_2_O and O_2_ (∼0.3 nm). While the TCPP molecule exhibits a sharp Soret band absorption at ∼415–430 nm that is associated to the excitation of porphyrin from the ground state to the S_2_ state, a significant broadening of absorption to 1,100 nm is observed for the SA-TCPP nanosheet (Figure 7e). This corresponds to an optical bandgap of 1.57 eV for the supramolecular SA-TCPP photocatalyst due to strong intermolecular π-π stacking interactions between porphyrin rings and the arisen excitonic coupling. Further, the derived band edge potentials of the SA-TCPP supramolecular photocatalyst reveal that conduction band minimum (CBM) of SA-TCPP permits the reduction of O_2_ to H_2_O_2_ (0.68 V vs RHE), whereas the valence band minimum (VBM) hinders the oxidation of water to H_2_O_2_ (Figure 7f). It is considered that the oxidation of the carboxylic group (R-COOH) on the SA-TCPP to peroxy acid groups (R-COOOH, 1.51 V vs RHE) is the dominant counter reaction for photocatalytic H_2_O_2_ production from O_2_ reduction, rather than the oxygen evolution from water oxidation, due to a low formation rate of H_2_O_2_ at RT and indirect electrochemical analysis. The thermally unstable peroxy intermediates are hydrolyzed back to RCOOH at a higher reaction temperature to complete the catalytic cycle, creating ^·^OH radicals that may also contribute to H_2_O_2_ formation. The temperature of an SA-TCPP-water suspension saturated with O_2_ reaches 328 K upon irradiation under a solar simulator, producing 2.36 mM H_2_O_2_ within 3 h that corresponds to a SCC efficiency of ∼1.2% in the absence of sacrificial reagents.

A better utilization of solar radiation can also be realized by employing up- and down- conversion luminescent materials, which absorb photons with inappropriate energy and emit photons with suitable energy that match the bandgap of the employed photocatalyst.^155,156^ This is practically important for certain catalytic chemical conversions that require specific photosensitizers and photocatalysts that only absorb a small portion of light from the solar spectrum.^157–160^ A wide range of inorganic and organic luminescent materials with characteristic absorption and emission provides a large tool box for the coupling with desired photocatalysts,^161^ with extra considerations on their stability under reaction conditions (i.e., solvent, pH, and temperature). Inspired by the leaf, a combination of luminescent solar concentrators (LSC) and continuous-flow photomicroreactors (LSC-PMs) is proposed by Cambié et al.^162^ Here, a fluorescent perylene bisimide-based dye (Lumogen F red 305, LR305) is chosen as the LSC luminophore due to its broad absorption spectrum, excellent photoluminescent quantum yield, and high photostability.^163^ The absorption covers a wide range up to 600 nm, emitting light from 600 nm to the infrared region that couples perfectly with the absorption spectrum of MB (Figure 7g), which serves as the photosensitizer for the singlet oxygen mediated cycloaddition of 9,10-diphenylanthracene. Further, the LSC-PM device is fabricated through a molding, casting, and plasma bonding process, employing the dye doped polydimethylsiloxane (PDMS) material (Sylgard 184, Figure 7h).^153^ The PDMS is selected as an ideal matrix for LSC-PM due to its high transparency, decent thermal and chemical stabilities, and moderate refractive index (1.41). The microchannel inside the LSC-PM enables the reaction mixture to experience not only direct sunlight but also the down-converted light from the fluorescent dye. Additionally, the microchannels also guide photons to travel shorter paths in the device, thus, reducing losses through internal reabsorption. A set of 50 × 50 × 3 mm^3^ serpentine LSC-PM devices (150 μL, 6 channels, 500 μm width × 1 mm height) made of LR305 doped PDMS with variable concentration of dye are employed to evaluate the performance of the system under solar simulator irradiation. A plateaued performance is observed at the concentration of LR305 in 100–200 ppm, possibly due to optimized light absorption and minimized quenching, fluorescence resonance energy transfer (FRET), and self-absorption losses. Remarkably, the LSC-PM device made of LR305 doped PDMS (200 ppm) shows a complete conversion of 9,10-diphenylanthracene under solar irradiation on a partly sunny summer day (323 W·m^–2^ on average) at different residence times (20, 15, and 10 s), which is significantly better than the nondoped reactor. Further, Zhao et al. from the same group have investigated the scalability of LSC-PMs via a numbering-up strategy.^153^ The molds can be fabricated via 3D printing, thus allowing fast optimization and mass-production of the system. Interestingly, reactors up to 32 parallel channels have been fabricated with an excellent flow distribution using a bifurcated flow distributor, which displays yields for the cycloaddition of 9,10-diphenylanthracene comparable to the single-channel device.

Masson et al. show the possibility of constructing a self-sufficient LSC-PM-based mini-plant for sunlight-driven photocatalytic oxidation of l-methionine (0.1 M) to produce sulfoxide (Figure 7i).^154^ Integrated solar panels are placed behind the LSC to utilize the transmitted fraction of the solar irradiation (600–1100 nm), providing electricity that enables the system to be fully operational off-grid. A responsive control system with a light sensor attached to the edge of the LSC-PM allows quick adjustment of the flow rate of the reagents to the light received by the reaction channels. A 40° angle is determined to be optimal to maximize the sunlight absorption by the LSC-PM, according to the ray-tracing Monte Carlo algorithm implemented in Python. A 0.47 × 0.47 m^2^ LSC-PM reactor in combination with a 0.38 × 0.51 m^2^ solar panel behind the reactor has been used for outdoor photocatalytic reaction on an intermittently cloudy day. Note that such a system requires additional solar panels placed next to the reactor to provide sufficient electricity required by the system (12–14 W), and an additional energy storage is needed for practical applications. A full conversion (>99%) with a constant high selectivity to the target product proves the efficiency of the autonomous off-grid system despite the cloudy weather, showing a maximum throughput of 17 mmol·h^–1^ l-methionine sulfoxide under strong direct irradiation conditions (∼60 klux) and a minimum throughput of 3 mmol·h^–1^ under very low irradiation (∼10 klux). It is estimated that by locating the mini-plant in regions closer to the equator (e.g., Townsville of Australia), a yearly productivity of 955 mol rose oxide can be reached, corresponding to a 150 m^2^ plant to reach the annual global demand of this chemical (390–650 kmol), rendering it a profitable process for commercialization. Alternatively, Wu et al. show the coupling of upconversion luminescent compounds with [Ru­(bpy)]^2+^ photosensitizer for solar-driven conversion of α-terpinene to ascaridole in flow.^164^ Here, the dye-sensitizer pair of diphenylanthracene (DPA) and 2,3,7,8,12,13,17,18 octaethyl-21*H*,23*H*-porphine palladium­(II) (PdOEP) is employed to convert longer-wavelength green light to emit blue light for photochemical transformations. The Q-band absorption of the PdOEP sensitizer is located in the green light region (centered at 516 and 548 nm), driving the photon upconversion process and transferring the photon energy to the emitter DPA molecule *via* a triplet energy transfer (TET) process. Two excited triplet DPAs produce a singlet excited DPA through a triplet–triplet annihilation (TTA) step, emitting blue light (peaks at ∼410 and 430 nm) and decaying to the ground state. The absorption of the [Ru­(bpy)]^2+^ overlaps well with the emission of DPA, thus can be excited to generate singlet oxygen from molecular triplet oxygen for the ene reaction. The flow reactor prepared using DPA- PdOEP doped urethane shows decent conversion efficiency of α-terpinene in a wide range of flow rates and irradiances, revealing the potential for some challenging redox reactions under solar irradiation. Nevertheless, the unwanted spectra region should be filtered for reactions that are sensitive to wavelength.^165,166^ A case study is the selective synthesis of azoaromatics and azoxyaromatics via photocatalytic reductive coupling of nitroaromatics by g-C_3_N_4_, which depends on the wavelength and yields unwanted hydroxylated azoaromatics in the presence of UV light.^108,126^

## 5 Conclusion and Perspective

In summary, we have reviewed recent developments on photocatalytic chemical conversions in continuous flow from the perspective of flow system design, utilization, and circulation of photocatalyst materials and potentially all solar-driven photocatalytic systems. An overwhelming interest is witnessed in recent years in attempting established batch photocatalytic reactions in continuous flow systems, aiming at (or showing determination for) scaling-up. Although industrial scale light-induced synthetic applications are still restricted in photochemical reactions for the production of pharmaceutical intermediates,^29,167–169^ a couple of evolutionary solutions have been evolved to accelerate the exploration of photocatalytic processes at a preparative scale. These solutions include the design, optimization, and fabrication of photocatalyst materials and innovations on the reaction systems with advanced materials for enhanced light utilization, mass transfer, and catalyst separation, as discussed in this perspective.

There is no doubt about the bright future of continuous flow photocatalytic chemical conversions, but time and patience are required. The critical challenge in scale-up of photocatalytic chemical conversion for industrialization is the conflict between cost and profit, which should be simply considered from the perspective of increasing the profit and reducing the cost (Figure 8a). This calls for both fundamental and applied research and development funded by public subsidies and industrial partners for the incubation of innovations in photocatalysis at different technology readiness levels (TRL). The implementation of pilot-scale photocatalytic plants should be encouraged for the assessment of the operation cost, profitability, and environmental impact.

**Figure 8:**
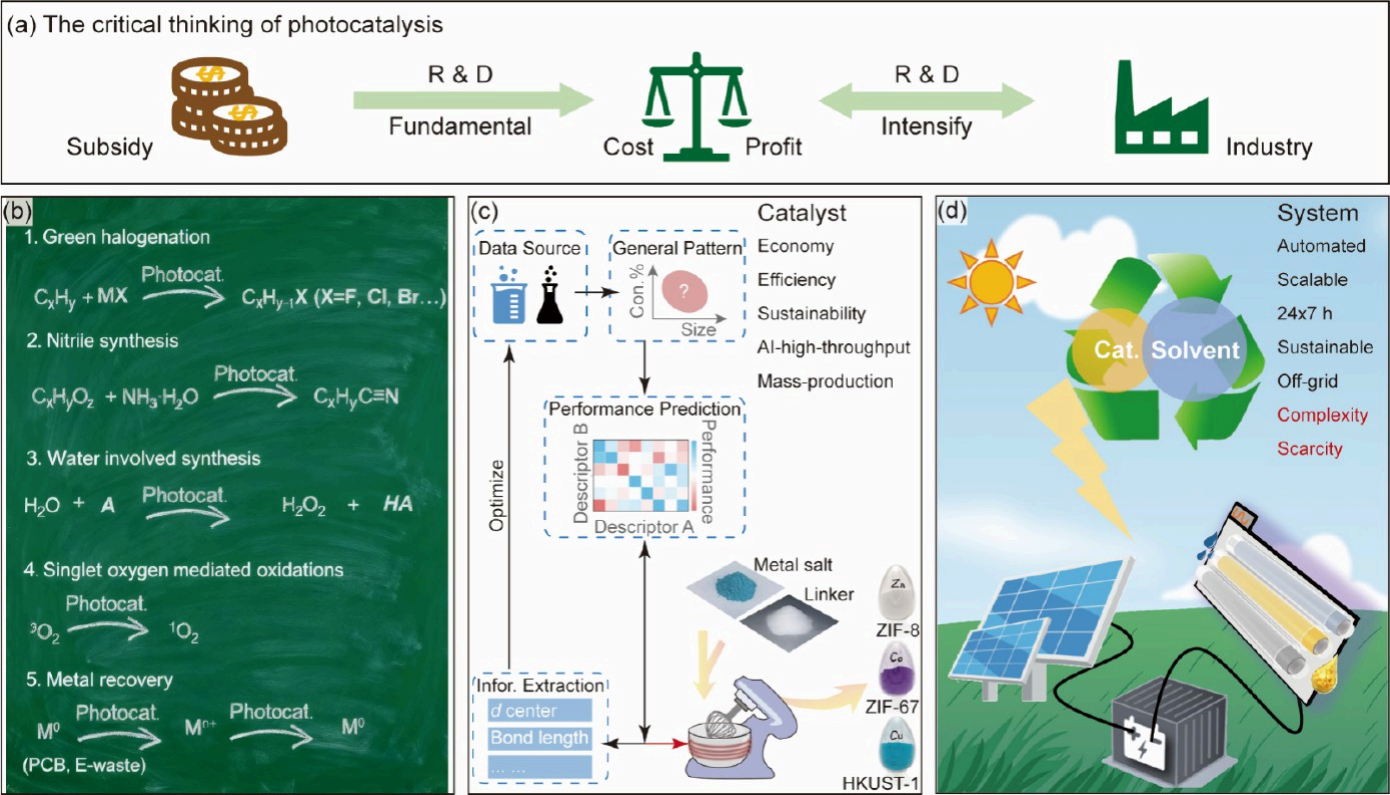
Outlook of photocatalytic chemical conversions. (a) The critical thinking of challenges in scaling-up for industrialization of photocatalysis, from the perspective of (b) Reaction types; (c) AI assisted high throughput catalyst design and mass production; and (d) Self-sustained devices and complete systems for 24 × 7 operation.

The selection of important chemical reactions is prioritized for profitable photocatalysis, especially for those reactions that can be realized by conventional homogeneous and heterogeneous catalysis but requires specialized reaction conditions, additives, handling, and storage. Chemicals with a bulk quantity and a high demand for conversion are also on the list. Within this scheme, we consider that halogenation employing inorganic salts,^170–172^ ammoxidation of hydrocarbons under atmospheric pressure using aqueous ammonia,^173,174^ selective hydrogenation using water as the hydrogen source,^175–178^ singlet oxygen mediated oxidation,^179,180^ and recovery of noble metals from electronic waste^181–183^ are representative profitable reactions (Figure 8b). However, the concentrations of reactants must be improved to a practical level (i.e., a few hundred millimolar to M), and the demand for solvents must be significantly reduced. This is essential to reach reasonable productivity and also crucial for late-stage product separation and purification that are energy and effort costly. The development of integrated multiscale computational fluid dynamics and radiation transport models may provide fundamental guidance of customized reactors and integrated systems to achieve this goal.

The design and optimization of photocatalyst materials are still the key for efficient and selective synthesis of value-added chemicals (Figure 8c). A usable photocatalyst for practical applications should be affordable, efficient, durable, sustainable, and can be produced at a large-scale. Therefore, the use of noble metals and complicated chemical precursors should be minimized, and if unavoidable (e.g., Pd for hydrogenation), should be cyclable.^184–192^ The integration of automated high-throughput platform, artificial intelligence (AI), and machine learning may accelerate the screening of photocatalyst materials.^186^ The construction of specialized databases encompassing catalyst properties, reactor parameters, and performance metrics could further boost the design of novel photocatalysts, the prediction of reaction outcomes, and the real-time optimization of operating conditions. We show that the mass-production of well-defined, highly crystalline MOFs photocatalyst at a kilogram scale can be realized by mechanochemistry in an hour.^187^ Additionally, real-time and *post-mortem* characterizations of photocatalysts used in flow still deserve investigations for better understanding of the deactivation process, thus providing feedbacks on immobilization and recovery of photocatalysts.^188^ The construction of pH and temperature-sensitive photocatalysts to achieve nondestructive recovery represents a new paradigm for enabling a circular chemistry.^189^

Once the reaction and the required photocatalyst are fixed, a complete flow system can be optimized by the perfection of individual devices with experts in chemical engineering (Figure 8d). It is essential to build a fully automated, saleable flow system that can be operated in a nonstop fashion.^190^ Additionally, a fully solar-powered system is preferred from the consideration of sustainability and minimized cost on electricity. This requires extra energy generation and storage (solar panels with Li-ion batteries) and artificial light (LED) subsystems, which provide electricity for control systems and accessories (e.g., pumps and sensors) during sunny daytime and for LED under insufficient irradiation for continuous operation. We have constructed a 0.5 m^2^ compact system employing an iron-based photocatalyst for this purpose.^191^ The application of fluorescent dye molecules for up- and down-conversion could be a supplement, but the development of efficient photocatalysts that absorb visible light is more crucial. Nevertheless, the system should also include subsystems for solvent circulation and regeneration; thus, low boiling point solvents might be preferred. Although a complete flow system offers enhanced controllability and scalability, its widespread adoption in industry remains limited due to the complexity in engineering and operation, high costs on fixed assets, and maintenance. We consider that 3D-printing is a promising solution for the design and fabrication of both prototypes and preparative scale flow reactors at an affordable expense.

## References

[1] Sambiagio C., Noël T. (2020). Flow Photochemistry: Shine Some Light
on Those Tubes!. Trends Chem..

[2] Zhang N., Gong W., Xiong Y. (2025). Modern organic
transformations:
heterogeneous
thermocatalysis or photocatalysis?. Chem. Soc.
Rev..

[3] Lim K. M., Khoo V., Ong W. J. (2025). Trash-to-Energy: Shedding Light on
Plastic and Biomass Valorization Through Artificial Photosynthesis
Towards Sustainability. Explor..

[4] Nakata K., Fujishima A. (2012). TiO2 photocatalysis:
Design and applications. J. Photochem. Photobiol.,
C.

[5] Mai H., Chen D., Tachibana Y., Suzuki H., Abe R., Caruso R. A. (2021). Developing sustainable,
high-performance perovskites
in photocatalysis: design strategies and applications. Chem. Soc. Rev..

[6] Cao H., Kong D., Yang L.-C., Chanmungkalakul S., Liu T., Piper J. L., Peng Z., Gao L., Liu X., Hong X., Wu J. (2022). Brønsted acid-enhanced
direct
hydrogen atom transfer photocatalysis for selective functionalization
of unactivated C­(*sp*
^3^)–H bonds. Nat. Synth..

[7] Chen L., Tang J., Song L.-N., Chen P., He J., Au C.-T., Yin S.-F. (2019). Heterogeneous
photocatalysis for
selective oxidation of alcohols and hydrocarbons. Appl. Catal., B.

[8] Hao L., Kang L., Huang H., Ye L., Han K., Yang S., Yu H., Batmunkh M., Zhang Y., Ma T. (2019). Surface-Halogenation-Induced Atomic-Site Activation and Local Charge
Separation for Superb CO_2_ Photoreduction. Adv. Mater..

[9] U.
Dighe S., Juliá F., Luridiana A., Douglas J. J., Leonori D. (2020). A photochemical dehydrogenative strategy
for aniline synthesis. Nature.

[10] Candish L., Collins K. D., Cook G. C., Douglas J. J., Gómez-Suárez A., Jolit A., Keess S. (2022). Photocatalysis in the Life Science
Industry. Chem. Rev..

[11] Cannalire R., Pelliccia S., Sancineto L., Novellino E., Tron G. C., Giustiniano M. (2021). Visible light
photocatalysis in the
late-stage functionalization of pharmaceutically relevant compounds. Chem. Soc. Rev..

[12] Chu S., Zhang B., Zhao X., Soo H. S., Wang F., Xiao R., Zhang H. (2022). Photocatalytic
Conversion of Plastic
Waste: From Photodegradation to Photosynthesis. Adv. Energy Mater..

[13] Keijer T., Bakker V., Slootweg J. C. (2019). Circular chemistry to enable a circular
economy. Nat. Chem..

[14] Lu Y., Luo R., Leng X., Huang Y., Jiang W., Besenbacher F., Li Y., Yin W., Su R. (2025). Water-Promoted Molar-Level Photocatalysis
and Spontaneous Product Separation with Near-Unity Quantum Efficiency. J. Am. Chem. Soc..

[15] Noël T., Buchwald S. L. (2011). Cross-coupling in
flow. Chem.
Soc. Rev..

[16] Yoshida J.-i., Takahashi Y., Nagaki A. (2013). Flash chemistry: flow chemistry that
cannot be done in batch. Chem. Commun..

[17] Zhang K., Li D., Cao H., Zhu Q., Trapalis C., Zhu P., Gao X., Wang C. (2021). Insights into
different dimensional MXenes for photocatalysis. Chem. Eng. J..

[18] Li K., Zhang S., Li Y., Fan J., Lv K. (2021). MXenes as
noble-metal-alternative co-catalysts in photocatalysis. Chin. J. Catal..

[19] Kant P., Liang S., Rubin M., Ozin G. A., Dittmeyer R. (2023). Low-cost photoreactors
for highly photon/energy-efficient solar-driven synthesis. Joule.

[20] Jähnisch K., Hessel V., Löwe H., Baerns M. (2004). Chemistry in Microstructured
Reactors. Angew. Chem., Int. Ed..

[21] Mason B. P., Price K. E., Steinbacher J. L., Bogdan A. R., McQuade D. T. (2007). Greener
Approaches to Organic Synthesis Using Microreactor Technology. Chem. Rev..

[22] Frost C. G., Mutton L. (2010). Heterogeneous catalytic
synthesis using microreactor
technology. Green Chem..

[23] Valera F. E., Quaranta M., Moran A., Blacker J., Armstrong A., Cabral J. T., Blackmond D. G. (2010). The Flow’s
the Thing...Or
Is It? Assessing the Merits of Homogeneous Reactions in Flask and
Flow. Angew. Chem., Int. Ed..

[24] Hayes H. L. D., Mallia C. J. (2024). Continuous Flow
Chemistry with Solids: A Review. Org. Process
Res. Dev..

[25] Baumann M., Moody T. S., Smyth M., Wharry S. (2020). A Perspective
on Continuous
Flow Chemistry in the Pharmaceutical Industry. Org. Process Res. Dev..

[26] West T. (2024). Developing
flow chemistry on scale. Nat. Synth..

[27] Guidi M., Seeberger P. H., Gilmore K. (2020). How to approach flow chemistry. Chem. Soc. Rev..

[28] Zhang J., Gong C., Zeng X., Xie J. (2016). Continuous flow chemistry:
New strategies for preparative inorganic chemistry. Coord. Chem. Rev..

[29] Raby-Buck S. E., Devlin J., Gupta P., Battilocchio C., Baumann M., Polyzos A., Slater A. G., Browne D. L. (2025). Continuous
flow chemistry for molecular synthesis. Nat.
Rev. Methods Primers.

[30] Plutschack M. B., Pieber B., Gilmore K., Seeberger P. H. (2017). The Hitchhiker’s
Guide to Flow Chemistry. Chem. Rev..

[31] Gilmore K., Seeberger P. H. (2014). Continuous Flow Photochemistry. Chem. Rec..

[32] Cambié D., Bottecchia C., Straathof N. J. W., Hessel V., Noël T. (2016). Applications
of Continuous-Flow Photochemistry in Organic Synthesis, Material Science,
and Water Treatment. Chem. Rev..

[33] Pomilla F. R., García-López E. I., Marcì G., Palmisano L., Parrino F. (2021). Heterogeneous photocatalytic materials
for sustainable formation of high-value chemicals in green solvents. Mater. Today Sustainability.

[34] Buglioni L., Raymenants F., Slattery A., Zondag S. D. A., Noël T. (2022). Technological
Innovations in Photochemistry for Organic Synthesis: Flow Chemistry,
High-Throughput Experimentation, Scale-up, and Photoelectrochemistry. Chem. Rev..

[35] Corbel S., Charles G., Becheikh N., Roques-Carmes T., Zahraa O. (2012). Modelling and design of microchannel
reactor for photocatalysis. Virtual Phys. Prototyping.

[36] Sohrabi S., Moraveji M. K., Iranshahi D. (2020). A review on the design and development
of photocatalyst synthesis and application in microfluidic reactors:
challenges and opportunities. Rev. Chem. Eng..

[37] Wang J., Yang C., Wang C., Han W., Zhu W. (2014). Photolytic
and photocatalytic degradation of micro pollutants in a tubular reactor
and the reaction kinetic models. Sep. Purif.
Technol..

[38] Grčić I., Li Puma G. (2017). Six-flux absorption-scattering models for photocatalysis
under wide-spectrum irradiation sources in annular and flat reactors
using catalysts with different optical properties. Appl. Catal., B.

[39] Portela R., Tessinari R. F., Suárez S., Rasmussen S. B., Hernández-Alonso M. D., Canela M. C., Ávila P., Sánchez B. (2012). Photocatalysis for Continuous Air
Purification in Wastewater
Treatment Plants: From Lab to Reality. Environ.
Sci. Technol..

[40] Raby-Buck S. E., Devlin J., Gupta P., Battilocchio C., Baumann M., Polyzos A., Slater A. G., Browne D. L. (2025). Continuous
flow chemistry for molecular synthesis. Nat.
Rev. Methods Primers.

[41] Sambiagio C., Noël T. (2020). Flow Photochemistry: Shine Some Light
on Those Tubes!. Trends Chem..

[42] Noël T., Zysman-Colman E. (2022). The promise
and pitfalls of photocatalysis for organic
synthesis. Chem. Catal..

[43] Nagornîi D., Raymenants F., Kaplaneris N., Noël T. (2024). C­(sp^3^)-H sulfinylation
of light hydrocarbons with sulfur dioxide via hydrogen
atom transfer photocatalysis in flow. Nat. Commun..

[44] Ovchinnikova E. V., Vernikovskaya N. V., Gribovskii A. G., Chumachenko V. A. (2021). Multichannel
microreactors for highly exothermic catalytic process: The influence
of thermal conductivity of reactor material and of transport phenomena
inside the channels on the process efficiency. Chem. Eng. J..

[45] Jang S., Jung B.-J., Kim M.-J., Lee W., Kim D.-P. (2019). Reaction-volume
dependent chemistry of highly selective photocatalytic reduction of
nitrobenzene. React. Chem. Eng..

[46] Mehta K. H., I Made R., Parkin I. P., Sankar G., Handoko A. D. (2025). A Paradigm
Shift: From Batch Processing to Flow Chemistry. Small.

[47] Zhao F., Chen Z., Fan W., Dou J., Li L., Guo X. (2020). Reactor optimization and process intensification of photocatalysis
for capillary-based PMMA LSC-photomicroreactors. Chem. Eng. J..

[48] Steiner A., Williams J. D., de Frutos O., Rincón J. A., Mateos C., Kappe C. O. (2020). Continuous photochemical
benzylic
bromination using in situ generated Br_2_: process intensification
towards optimal PMI and throughput. Green Chem..

[49] Cao Z., Ji M., Wang X., Wu X., Li Y., Zhu C. (2022). Metal-free
photo-induced heteroarylations of C-H and C-C bonds of alcohols by
flow chemistry. Green Chem..

[50] Corcoran E. B., McMullen J. P., Lévesque F., Wismer M. K., Naber J. R. (2020). Photon
Equivalents as a Parameter for Scaling Photoredox Reactions in Flow:
Translation of Photocatalytic C-N Cross-Coupling from Lab Scale to
Multikilogram Scale. Angew. Chem., Int. Ed..

[51] Elliott L. D., Knowles J. P., Stacey C. S., Klauber D. J., Booker-Milburn K. I. (2018). Using batch
reactor results to calculate optimal flow rates for the scale-up of
UV photochemical reactions. React. Chem. Eng..

[52] Steiner A., de Frutos O., Rincón J. A., Mateos C., Williams J. D., Kappe C. O. (2021). N-Chloroamines
as substrates for metal-free photochemical
atom-transfer radical addition reactions in continuous flow. React. Chem. Eng..

[53] Vidyacharan S., Ramanjaneyulu B. T., Jang S., Kim D. P. (2019). Continuous-Flow
Visible Light Organophotocatalysis for Direct Arylation of 2H-Indazoles:
Fast Access to Drug Molecules. ChemSusChem.

[54] Bottecchia C., Lévesque F., McMullen J. P., Ji Y., Reibarkh M., Peng F., Tan L., Spencer G., Nappi J., Lehnherr D., Narsimhan K., Wismer M. K., Chen L., Lin Y., Dalby S. M. (2022). Manufacturing Process Development for Belzutifan, Part
2: A Continuous Flow Visible-Light-Induced Benzylic Bromination. Org. Process Res. Dev..

[55] Horie T., Sumino M., Tanaka T., Matsushita Y., Ichimura T., Yoshida J.-i. (2010). Photodimerization
of Maleic Anhydride
in a Microreactor Without Clogging. Org. Process
Res. Dev..

[56] Feng S., Su R. (2024). Synthetic Chemistry
in Flow: From Photolysis & Homogeneous Photocatalysis
to Heterogeneous Photocatalysis. ChemSusChem.

[57] Wu K.-J., Nappo V., Kuhn S. (2015). Hydrodynamic Study of Single- and
Two-Phase Flow in an Advanced-Flow Reactor. Ind. Eng. Chem. Res..

[58] Mandigma M. J. P., Žurauskas J., MacGregor C. I., Edwards L. J., Shahin A., d’Heureuse L., Yip P., Birch D. J. S., Gruber T., Heilmann J., John M. P., Barham J. P. (2022). An organophotocatalytic late-stage N-CH_3_ oxidation of trialkylamines to N-formamides with O_2_ in
continuous flow. Chem. Sci..

[59] Brzozowski M., O’Brien M., Ley S. V., Polyzos A. (2015). Flow Chemistry: Intelligent
Processing of Gas–Liquid Transformations Using a Tube-in-Tube
Reactor. Acc. Chem. Res..

[60] Cortes-Quiroz C. A., Azarbadegan A., Zangeneh M. (2017). Effect of channel aspect ratio of
3-D T-mixer on flow patterns and convective mixing for a wide range
of Reynolds number. Sens. Actuators, B.

[61] Laudadio G., Govaerts S., Wang Y., Ravelli D., Koolman H. F., Fagnoni M., Djuric S. W., Noël T. (2018). Selective
C­(sp^3^)-H Aerobic Oxidation Enabled by Decatungstate Photocatalysis
in Flow. Angew. Chem., Int. Ed..

[62] Mendoza
Suarez F., Tatarchuk B. (2025). Comparative economic analysis of
batch vs. continuous manufacturing in catalytic heterogeneous processes:
impact of catalyst activity maintenance and materials costs on total
costs of manufacturing in the production of fine chemicals and pharmaceuticals. J. Flow Chem..

[63] Straathof N. J. W., Su Y., Hessel V., Noël T. (2016). Accelerated
gas-liquid visible light photoredox catalysis with continuous-flow
photochemical microreactors. Nat. Protoc..

[64] Lévesque F., Seeberger P. H. (2012). Continuous-Flow Synthesis of the Anti-Malaria Drug
Artemisinin. Angew. Chem., Int. Ed..

[65] Kupracz L., Kirschning A. (2013). Multiple Organolithium Generation in the Continuous
Flow Synthesis of Amitriptyline. Adv. Synth.
Catal..

[66] Pieber B., Shalom M., Antonietti M., Seeberger P. H., Gilmore K. (2018). Continuous Heterogeneous Photocatalysis in Serial Micro-Batch
Reactors. Angew. Chem., Int. Ed..

[67] Dong Z., Zondag S. D. A., Schmid M., Wen Z., Noël T. (2022). A meso-scale
ultrasonic milli-reactor enables gas-liquid-solid photocatalytic reactions
in flow. Chem. Eng. J..

[68] Yu Z., Zhao J., Wu Y., Guo L., Ma C., Zhu H., Li J., Duan L., Liu Z., Sun H., Zhao G., Meng Q. (2023). Sustainable and Continuous
Flow Preparation
of High-Concentration H_2_O_2_ by Visible-Light-Activated
Molecular Oxygen Oxidation of Lower Alcohols. ACS Sustainable Chem. Eng..

[69] O’Brien M., Taylor N., Polyzos A., Baxendale I. R., Ley S. V. (2011). Hydrogenation in flow: Homogeneous
and heterogeneous
catalysis using Teflon AF-2400 to effect gas-liquid contact at elevated
pressure. Chem. Sci..

[70] Pastre J. C., Browne D. L., O’Brien M., Ley S. V. (2013). Scaling Up of Continuous
Flow Processes with Gases Using a Tube-in-Tube Reactor: Inline Titrations
and Fanetizole Synthesis with Ammonia. Org.
Process Res. Dev..

[71] Koos P., Gross U., Polyzos A., O’Brien M., Baxendale I., Ley S. V. (2011). Teflon AF-2400 mediated
gas–liquid
contact in continuous flow methoxycarbonylations and in-line FTIR
measurement of CO concentration. Org. Biomol.
Chem..

[72] Kouridaki A., Huvaere K. (2017). Singlet oxygen
oxidations in homogeneous continuous
flow using a gas-liquid membrane reactor. React.
Chem. Eng..

[73] Yang L., Jensen K. F. (2013). Mass Transport and Reactions in the Tube-in-Tube Reactor. Org. Process Res. Dev..

[74] Doyle B. J., Gutmann B., Bittel M., Hubler T., Macchi A., Roberge D. M. (2020). Handling of Solids
and Flow Characterization in a Baffleless
Oscillatory Flow Coil Reactor. Ind. Eng. Chem.
Res..

[75] Roibu A., Van Gerven T., Kuhn S. (2020). Photon Transport and
Hydrodynamics
in Gas-Liquid Flows Part 1: Characterization of Taylor Flow in a Photo
Microreactor. ChemPhotoChem..

[76] Roibu A., Horn C. R., Van Gerven T., Kuhn S. (2020). Photon Transport and
Hydrodynamics in Gas-Liquid Flow Part 2: Characterization of Bubbly
Flow in an Advanced-Flow Reactor. ChemPhotoChem..

[77] Li X., Zhang X. J., Guo W. L., Huang Y., Cai T. (2023). Shape-Controlled
synthesis of conjugated microporous polymer nanotubes and their implementation
in continuous flow polymerization. Chem. Eng.
J..

[78] Shang Q., Guo M., Zhang J., Liu M., Lin K., Li H., Cui G., Shi X., Tang B. (2024). Gas-liquid-soild triphasic continuous
flow microreactor for improving homogeneous distribution of solid
composites in heterogeneous photocatalytic degradation progress. Sep. Purif. Technol..

[79] Rosso C., Gisbertz S., Williams J. D., Gemoets H. P. L., Debrouwer W., Pieber B., Kappe C. O. (2020). An oscillatory plug
flow photoreactor
facilitates semi-heterogeneous dual nickel/carbon nitride photocatalytic
C-N couplings. React. Chem. Eng..

[80] Vural
Gürsel I., Noël T., Wang Q., Hessel V. (2015). Separation/recycling
methods for homogeneous transition metal catalysts in continuous flow. Green Chem..

[81] Maity K., Sau S., Banerjee F., Samanta S. K. (2024). Heterogenization of Homogeneous Donor–Acceptor
Conjugated Polymers for Efficient Photooxidation: An Approach Toward
Sustainable and Recyclable Photocatalysis. ACS
Appl. Mater. Interfaces.

[82] Mak C. H., Han X., Du M., Kai J.-J., Tsang K. F., Jia G., Cheng K.-C., Shen H.-H., Hsu H.-Y. (2021). Heterogenization
of homogeneous photocatalysts utilizing synthetic and natural support
materials. J. Mater. Chem. A.

[83] Zhang J.-H., Gong Y.-N., Wang H.-J., Wang Y.-C., Yang W., Mei J.-H., Zhong D.-C., Lu T.-B. (2022). Ordered heterogeneity
of molecular photosensitizer toward enhanced photocatalysis. Proc. Natl. Acad. Sci. U.S.A..

[84] Rossi S., Herbrik F., Resta S., Puglisi A. (2022). Supported Eosin Y as
a Photocatalyst for C-H Arylation of Furan in Batch and Flow. Molecules.

[85] De
Vos A., Lejaeghere K., Muniz Miranda F., Stevens C. V., Van Der
Voort P., Van Speybroeck V. (2019). Electronic properties of heterogenized
Ru­(ii) polypyridyl photoredox complexes on covalent triazine frameworks. J. Mater. Chem. A.

[86] Cantillo D., Kappe C. O. (2014). Immobilized Transition
Metals as Catalysts for Cross-Couplings
in Continuous Flow—A Critical Assessment of the Reaction Mechanism
and Metal Leaching. ChemCatChem..

[87] Wen Z., Pintossi D., Nuño M., Noël T. (2022). Membrane-based
TBADT recovery as a strategy to increase the sustainability of continuous-flow
photocatalytic HAT transformations. Nat. Commun..

[88] Yang H., Xu J., Cao H., Wu J., Zhao D. (2023). Recovery of homogeneous
photocatalysts by covalent organic framework membranes. Nat. Commun..

[89] Hall J. F. B., Han X., Poliakoff M., Bourne R. A., George M. W. (2012). Maximising
the efficiency of continuous photo-oxidation with singlet oxygen in
supercritical CO_2_ by use of fluorous biphasic catalysis. Chem. Commun..

[90] Shao C., Yu X., Ji Y., Xu J., Yan Y., Hu Y., Li Y., Huang W., Li Y. (2024). Perfluoroalkyl-modified covalent
organic frameworks for continuous photocatalytic hydrogen peroxide
synthesis and extraction in a biphasic fluid system. Nat. Commun..

[91] Fangueiro D., Bermond A., Santos E., Carapuça H., Duarte A. (2002). Heavy metal mobility assessment in sediments based
on a kinetic approach of the EDTA extraction: search for optimal experimental
conditions. Anal. Chim. Acta.

[92] Singh S., Kaur M., Bajwa B. S., Kaur I. (2022). Salicylaldehyde and
3-hydroxybenzoic acid grafted NH_2_-MCM-41: Synthesis, characterization
and application as U­(VI) scavenging adsorbents using batch mode, column
and membrane systems. J. Mol. Liq..

[93] Fuchs M., Goessler W., Pilger C., Kappe C. O. (2010). Mechanistic Insights
into Copper­(I)-Catalyzed Azide-Alkyne Cycloadditions using Continuous
Flow Conditions. Adv. Synth. Catal..

[94] Goel S., Pant K. K., Nigam K. D. P. (2009). Extraction of nickel from spent catalyst
using fresh and recovered EDTA. J. Hazard. Mater..

[95] Cánovas C. R., Chapron S., Arrachart G., Pellet-Rostaing S. (2019). Leaching of
rare earth elements (REEs) and impurities from phosphogypsum: A preliminary
insight for further recovery of critical raw materials. J. Clean. Prod..

[96] Vural
Gürsel I., Aldiansyah F., Wang Q., Noël T., Hessel V. (2015). Continuous metal scavenging and coupling to one-pot
copper-catalyzed azide-alkyne cycloaddition click reaction in flow. Chem. Eng. J..

[97] Peeva L., Da Silva Burgal J., Heckenast Z., Brazy F., Cazenave F., Livingston A. (2016). Continuous
Consecutive Reactions with Inter-Reaction
Solvent Exchange by Membrane Separation. Angew.
Chem., Int. Ed..

[98] O’Neal E. J., Lee C. H., Brathwaite J., Jensen K. F. (2015). Continuous Nanofiltration
and Recycle of an Asymmetric Ketone Hydrogenation Catalyst. ACS Catal..

[99] Marchetti P., Jimenez Solomon M. F., Szekely G., Livingston A. G. (2014). Molecular
Separation with Organic Solvent Nanofiltration: A Critical Review. Chem. Rev..

[100] Janssen M., Müller C., Vogt D. (2011). Recent advances in
the recycling of homogeneous catalysts using membrane separation. Green Chem..

[101] Masliy V., Guillaume S. M., Fischmeister C., Carpentier J.-F. (2025). Molecular
weight enlargement of homogeneous catalysts
for enhanced recovery via organic solvent nanofiltration: A critical
review. Coord. Chem. Rev..

[102] Vandezande P., Gevers L. E. M., Vankelecom I. F. J. (2008). Solvent
resistant nanofiltration: separating on a molecular level. Chem. Soc. Rev..

[103] SolSep BV. Solvent Resistent Membranes, 2018; https://www.solsep.com/SRM.htm.

[104] Worrall D. R., Abdel-Shafi A. A., Wilkinson F. (2001). Factors Affecting
the Rate of Decay of the First Excited Singlet State of Molecular
Oxygen O_2_(a^1^Δ_g_) in Supercritical
Fluid Carbon Dioxide. J. Phys. Chem. A.

[105] Ye J., Zhang D., Salli S., Li Y., Han F., Mai Y., Rosei F., Li Y., Yang Y., Besenbacher F., Niemantsverdriet H., Richards E., Su R. (2023). Heterogeneous Photocatalytic
Recycling of FeX_2_/FeX_3_ for Efficient Halogenation
of C-H Bonds Using NaX. Angew. Chem., Int. Ed..

[106] Yang W., Feng S., Zhang X., Wang Y., Li C., Zhang L., Zhao J., Gurzadyan G. G., Tao S. (2021). Bodipy-Containing Porous Microcapsules for Flow Heterogeneous Photocatalysis. ACS Appl. Mater. Interfaces.

[107] Yang C., Li R., Zhang K. A. I., Lin W., Landfester K., Wang X. (2020). Heterogeneous photoredox
flow chemistry
for the scalable organosynthesis of fine chemicals. Nat. Commun..

[108] Liu W., Zhang D., Yue H., Li Y., Rosei F., Liu D., Su R. (2023). Immobilizing graphitic
carbon nitride on porous silica
via hydrogen bonds for photocatalytic flow synthesis of azoxybenzene. Chem. Eng. J..

[109] Li Y., Zhang D., Ye J., Mai Y., Wang C., Yang Y., Li Y., Besenbacher F., Niemantsverdriet H., Rosei F., Pan F., Su R. (2023). A Modular
Tubular Flow System with Replaceable Photocatalyst Membranes for Scalable
Coupling and Hydrogenation. Angew. Chem., Int.
Ed..

[110] Krivec M., Žagar K., Suhadolnik L., Čeh M., Dražić G. (2013). Highly Efficient TiO_2_-Based Microreactor for Photocatalytic Applications. ACS Appl. Mater. Interfaces.

[111] Wang T., Hu J., Ouyang R., Wang Y., Huang Y., Hu S., Li W.-X. (2024). Nature
of metal-support
interaction for metal catalysts on oxide supports. Science.

[112] Xu M., Bing Q., Tu Y., Zhang Y., Zhang M., Cai Y., Li J., Meng X., Zhu J., Yu L., Deng D. (2024). Full-Spectrum
Light-Harvesting Solar Thermal Electrocatalyst Boosts
Oxygen Evolution. Angew. Chem., Int. Ed..

[113] Wang J., Kong Y., Ren L., Shuang Y., Hong C., Ye Q., Wang S., Ma Z., Wang F., Cao T., Jian J., Wang H. (2026). Dual Dynamics
Engineering in COF@MOF Enabled by Laser-Embedded Metallic Clusters
for Efficient Photocatalytic Hydrogenation. Adv. Mater..

[114] Ma X.-L., Ma L.-H., Guo S., Zhang Z.-M., Lu T.-B. (2025). Identifying the Key Photosensitizing Factors over Metal-Organic Frameworks
for Selective Control of ^1^O_2_ and O_2·_
^–^ Generation. Angew. Chem.,
Int. Ed..

[115] Ma X.-L., Shi W.-X., Guo S., Zhao Q.-P., Lin W., Lu T.-B., Zhang Z.-M. (2025). Gram-Scale Green-Synthesis of High
Purity Pinacols and Amides by Continuous Tandem Photocatalysis via
a Negative Carbon Emission Process. Adv. Mater..

[116] Xu J., Chong M., Li W., Zhu E., Jin H., Liu L., Ren Y., Zhu Y. (2025). Guiding electron transfer for selective
C_2_H_6_ photoproduction from CO_2_. Chem..

[117] Reinhard J., Urban P., Bell S., Carpenter D., Sagoo M. S. (2024). Automatic data-driven design and 3D printing of custom
ocular prostheses. Nat. Commun..

[118] Pessoa S., Guimarães A. S., Lucas S. S., Simões N. (2021). 3D printing
in the construction industry—A systematic review of the thermal
performance in buildings. RENEW SUST ENERG REV.

[119] Bai S.-w., Mei H., Zhang M.-g., Zhou S.-x., Yan Y.-k., Cheng L.-f., Zhang L.-t., Lu J. (2023). 3D printing
assemble technology toward advanced photocatalysis. Mater. Today Nano.

[120] Zhang L., Zhu Z., Liu B., Li C., Yu Y., Tao S., Li T. (2019). Fluorescent Fluid in 3D-Printed Microreactors
for the Acceleration of Photocatalytic Reactions. Adv. Sci..

[121] Formlabs. Guide to Stereolithography (SLA) 3D Printing, 2020; https://formlabs.com/blog/ultimate-guide-to-stereolithography-sla-3d-printing/.

[122] Sopha H., Kashimbetova A., Baudys M., Chennam P. K., Sepúlveda M., Rusek J., Kolibalova E., Celko L., Montufar E. B., Krysa J., Macak J. M. (2023). Flow-through
Gas Phase Photocatalysis Using TiO_2_ Nanotubes on Wirelessly
Anodized 3D-Printed TiNb Meshes. Nano Lett..

[123] Díez A. M., Moreira F. C., Marinho B. A., Espíndola J. C. A., Paulista L. O., Sanromán M. A., Pazos M., Boaventura R. A. R., Vilar V. J. P. (2018). A step forward in heterogeneous photocatalysis: Process
intensification by using a static mixer as catalyst support. Chem. Eng. J..

[124] Luo J., Ruta V., Kwon I. S., Albertazzi J., Allasia N., Nevskyi O., Busini V., Moscatelli D., Vilé G. (2024). Fabricating a Structured Single-Atom Catalyst via High-Resolution
Photopolymerization 3D Printing. Adv. Funct.
Mater..

[125] Zhakeyev A., Jones M. C., Thomson C. G., Tobin J. M., Wang H., Vilela F., Xuan J. (2021). Additive manufacturing
of intricate and inherently photocatalytic flow reactor components. Addit. Manuf..

[126] Dai Y., Li C., Shen Y., Lim T., Xu J., Li Y., Niemantsverdriet H., Besenbacher F., Lock N., Su R. (2018). Light-tuned selective photosynthesis
of azo- and azoxy-aromatics using graphitic C_3_N_4_. Nat. Commun..

[127] Spasiano D., Marotta R., Malato S., Fernandez-Ibañez P., Di Somma I. (2015). Solar photocatalysis: Materials, reactors, some commercial,
and pre-industrialized applications. A comprehensive approach. Appl. Catal. B Environ.

[128] Cambié D., Noël T. (2018). Solar Photochemistry
in Flow. Top. Curr. Chem..

[129] Valencia-Valero L. C., Simbaña K. A., Eleraky J. E., Hapońska M., Puga A. (2025). Demonstration of hydrogen
production from food industry wastewaters
by solar photoreforming: From the laboratory to outdoors operation
in panel reactors. Chem. Eng. J..

[130] Wang J., Ran G., Gao J., Li D., Waterhouse G. I. N., Shi R., Zhang W., Tang J., Wu L. Z., Zhao Y., Zhang T. (2025). Solar-Driven
Conversion
of Nitrogen and Water to Solid Fertilizer in an Outdoor 1 m^2^ Panel Reactor. Adv. Mater..

[131] Endres C. H., Roth A., Brück T. B. (2018). Modeling
Microalgae Productivity in Industrial-Scale Vertical Flat Panel Photobioreactors. Environ. Sci. Technol..

[132] Zhao Y., Ding C., Zhu J., Qin W., Tao X., Fan F., Li R., Li C. (2020). A Hydrogen
Farm Strategy
for Scalable Solar Hydrogen Production with Particulate Photocatalysts. Angew.Chem. Int. Ed..

[133] Fu H., Wu Y., Guo Y., Sakurai T., Zhang Q., Liu Y., Zheng Z., Cheng H., Wang Z., Huang B., Wang Q., Domen K., Wang P. (2025). A scalable solar-driven
photocatalytic system for separated H_2_ and O_2_ production from water. Nat. Commun..

[134] Nishiyama H., Yamada T., Nakabayashi M., Maehara Y., Yamaguchi M., Kuromiya Y., Nagatsuma Y., Tokudome H., Akiyama S., Watanabe T., Narushima R., Okunaka S., Shibata N., Takata T., Hisatomi T., Domen K. (2021). Photocatalytic solar hydrogen production from water on a 100-m^2^ scale. Nature.

[135] Chi W., Dong Y., Liu B., Pan C., Zhang J., Zhao H., Zhu Y., Liu Z. (2024). A photocatalytic
redox
cycle over a polyimide catalyst drives efficient solar-to-H_2_O_2_ conversion. Nat. Commun..

[136] Jung C., Funken K.-H., Ortner J. (2005). PROPHIS: parabolic
trough-facility for organic photochemical syntheses in sunlight. Photochem. Photobiol. Sci..

[137] Kar S., Kim D., Bin Mohamad
Annuar A., Sarma B. B., Stanton M., Lam E., Bhattacharjee S., Karak S., Greer H. F., Reisner E. (2025). Direct air
capture of CO_2_ for solar fuel
production in flow. Nat. Energy.

[138] Maeda K., Teramura K., Lu D., Takata T., Saito N., Inoue Y., Domen K. (2006). Characterization
of
Rh-Cr Mixed-Oxide Nanoparticles Dispersed on (Ga_1‑x_Zn_x_)­(N_1‑x_O_x_) as a Cocatalyst
for Visible-Light-Driven Overall Water Splitting. J. Phys. Chem. B.

[139] Yang N., Yin Z., Chen Z., Gao C., Cao Z., Zheng Y., Pan Z., Cao H., Ye S., Xiong Y. (2025). Solar-Driven Massive
Production of Dimerized Imine in Aqueous Phase
via an Atomically Engineered Photocatalyst. Angew. Chem., Int. Ed..

[140] Wang X., Liu B., Ma S., Zhang Y., Wang L., Zhu G., Huang W., Wang S. (2024). Induced dipole
moments in amorphous ZnCdS catalysts facilitate photocatalytic H_2_ evolution. Nat. Commun..

[141] Grosjean A., Soum-Glaude A., Thomas L. (2021). Replacing silver by
aluminum in solar mirrors by improving solar reflectance with dielectric
top layers. Sustainable Mater.Technol..

[142] Newport. Introduction to Solar Radiation, 2013; https://www.newport.com.cn/t/introduction-to-solar-radiation.

[143] Oelgemöller M., Jung C., Ortner J., Mattay J., Zimmermann E. (2005). Green photochemistry: solar photooxygenations
with
medium concentrated sunlight. Green Chem..

[144] Malato S., Blanco J., Vidal A., Richter C. (2002). Photocatalysis
with solar energy at a pilot-plant scale: an overview. Appl. Catal. B Environ.

[145] Kositzi M., Poulios I., Malato S., Caceres J., Campos A. (2004). Solar photocatalytic treatment of synthetic municipal
wastewater. Water Res..

[146] Radjenović J., Sirtori C., Petrović M., Barceló D., Malato S. (2009). Solar photocatalytic degradation
of persistent pharmaceuticals at pilot-scale: Kinetics and characterization
of major intermediate products. Appl. Catal.,
B.

[147] Matos J., Miralles-Cuevas S., Ruíz-Delgado A., Oller I., Malato S. (2017). Development of TiO_2_-C
photocatalysts for solar treatment of polluted water. Carbon.

[148] Nahim-Granados S., Rivas-Ibáñez G., Antonio
Sánchez Pérez J., Oller I., Malato S., Polo-López M. I. (2020). Fresh-cut wastewater reclamation: Techno-Economical
assessment of solar driven processes at pilot plant scale. Appl. Catal., B.

[149] Spasiano D., del Pilar Prieto Rodriguez L., Olleros J. C., Malato S., Marotta R., Andreozzi R. (2013). TiO_2_/Cu­(II) photocatalytic production of benzaldehyde from benzyl alcohol
in solar pilot plant reactor. Appl. Catal.,
B.

[150] Barzegar M. H., Sabzehmeidani M. M., Ghaedi M., Avargani V. M., Moradi Z., Roy V. A. L., Heidari H. (2021). S-scheme heterojunction
g-C_3_N_4_/TiO_2_ with enhanced photocatalytic
activity for degradation of a binary mixture of cationic dyes using
solar parabolic trough reactor. Chem. Eng. Res.
Des..

[151] Zhou Q., Guo Y., Zhu Y. (2023). Photocatalytic sacrificial
H_2_ evolution dominated by micropore-confined exciton transfer
in hydrogen-bonded organic frameworks. Nat.
Catal..

[152] Zhang Y., Pan C., Bian G., Xu J., Dong Y., Zhang Y., Lou Y., Liu W., Zhu Y. (2023). H_2_O_2_ generation from O_2_ and H_2_O on a near-infrared absorbing porphyrin supramolecular photocatalyst. Nat. Energy.

[153] Zhao F., Cambié D., Janse J., Wieland E. W., Kuijpers K. P. L., Hessel V., Debije M. G., Noël T. (2018). Scale-up of
a Luminescent Solar Concentrator-Based Photomicroreactor via Numbering-up. ACS Sustainable Chem. Eng..

[154] Masson T. M., Zondag S. D. A., Kuijpers K. P. L., Cambié D., Debije M. G., Noël T. (2021). Development
of an Off-Grid Solar-Powered
Autonomous Chemical Mini-Plant for Producing Fine Chemicals. ChemSusChem.

[155] Richards B. S., Hudry D., Busko D., Turshatov A., Howard I. A. (2021). Photon Upconversion for Photovoltaics
and Photocatalysis:
A Critical Review. Chem. Rev..

[156] Cates E. L., Chinnapongse S. L., Kim J.-H., Kim J.-H. (2012). Engineering
Light: Advances in Wavelength Conversion Materials for Energy and
Environmental Technologies. Environ. Sci. Technol..

[157] Huang N.-Y., He H., Liu S., Zhu H.-L., Li Y.-J., Xu J., Huang J.-R., Wang X., Liao P.-Q., Chen X.-M. (2021). Electrostatic Attraction-Driven
Assembly
of a Metal-Organic Framework with a Photosensitizer Boosts Photocatalytic
CO_2_ Reduction to CO. J. Am. Chem.
Soc..

[158] Muniz C. N., Archer C. A., Applebaum J. S., Alagaratnam A., Schaab J., Djurovich P. I., Thompson M. E. (2023). Two-Coordinate Coinage Metal Complexes as Solar Photosensitizers. J. Am. Chem. Soc..

[159] Takata T., Domen K. (2019). Particulate Photocatalysts
for Water
Splitting: Recent Advances and Future Prospects. ACS Energy Lett..

[160] Wang Y., Vogel A., Sachs M., Sprick R. S., Wilbraham L., Moniz S. J. A., Godin R., Zwijnenburg M. A., Durrant J. R., Cooper A. I., Tang J. (2019). Current understanding
and challenges of solar-driven hydrogen generation using polymeric
photocatalysts. Nat. Energy.

[161] Zhuo Y., Brgoch J. (2021). Opportunities for Next-Generation
Luminescent Materials through Artificial Intelligence. J. Phys. Chem. Lett..

[162] Cambié D., Zhao F., Hessel V., Debije M. G., Noël T. (2017). A Leaf-Inspired Luminescent Solar
Concentrator for
Energy-Efficient Continuous-Flow Photochemistry. Angew. Chem., Int. Ed..

[163] Slooff L. H., Bende E. E., Burgers A. R., Budel T., Pravettoni M., Kenny R. P., Dunlop E. D., Büchtemann A. (2008). A luminescent
solar concentrator with 7.1% power conversion efficiency. Phys. Status Solidi RRL.

[164] Wu M., Moser B. A., Steeves T. M., Figueroa A., Wallace B. M., Kim S. T., Esser-Kahn A. P., Steinhardt R. C. (2019). Photon
upconversion for the enhancement of microfluidic photochemical synthesis. RSC Adv..

[165] Romero N. A., Nicewicz D. A. (2016). Organic Photoredox
Catalysis. Chem. Rev..

[166] Xiao J., Hisatomi T., Domen K. (2023). Narrow-Band-Gap
Particulate
Photocatalysts for One-Step-Excitation Overall Water Splitting. Acc. Chem. Res..

[167] Gutmann B., Cantillo D., Kappe C. O. (2015). Continuous-Flow
Technology—A Tool for the Safe Manufacturing of Active Pharmaceutical
Ingredients. Angew. Chem., Int. Ed..

[168] Zhao D. (2013). Updated Applications
of Flow Chemistry in Pharmaceutical Synthesis. Chin. J. Org. Chem..

[169] Sumino Y., Fukuyama T. (2012). Role of Flow Microreactors for Pharmaceutical
Production: Screening of Reaction Conditions and Sample Preparation. J. Synth. Org. Chem. Jpn..

[170] Cotty S., Jeon J., Elbert J., Jeyaraj V. S., Mironenko A. V., Su X. (2022). Electrochemical recycling
of homogeneous
catalysts. Sci. Adv..

[171] Parrino F., Camera Roda G., Loddo V., Palmisano L. (2016). Elemental
Bromine Production by TiO_2_ Photocatalysis and/or Ozonation. Angew. Chem., Int. Ed..

[172] Markushyna Y., Schüßlbauer C. M., Ullrich T., Guldi D. M., Antonietti M., Savateev A. (2021). Chromoselective Synthesis
of Sulfonyl Chlorides and Sulfonamides with Potassium Poly­(heptazine
imide) Photocatalyst. Angew. Chem., Int. Ed..

[173] Xian C., He J., He Y., Nie J., Yuan Z., Sun J., Martens W. N., Qin J., Zhu H.-Y., Zhang Z. (2022). High Nitrile
Yields of Aerobic Ammoxidation
of Alcohols Achieved by Generating ^·^O_2_
^–^ and Br^·^ Radicals over Iron-Modified
TiO_2_ Photocatalysts. J. Am. Chem.
Soc..

[174] Zhang D., Fan X., Feng S., Mai Y., Cai N., Xie Y., Li Y., Lin H., Su R. (2025). Selective
photo-conversion of benzaldehyde with ammonia tuned by metal nanoparticles. Cell Rep. Phys. Sci..

[175] Xu Q., Cai N., Maliutina K., Hu C., Zhang D., Huang Y., Mai Y., Rosei F., Li Y., Besenbacher F., Richards E., Lim T., Su R. (2025). Photocatalytic
Partial Water Dissociation by Protonated Carbon Nitride for Hydrogenation
Reactions. Angew. Chem., Int. Ed..

[176] Zhao E., Morales-Vidal J., Yang Y., Mitchell S., Zhu Y., Hu Z., Chen J.-M., Haw S.-C., Chan T.-S., Fan Z., Wang Z.-J., López N., Pérez-Ramírez J., Chen Z. (2025). Diatomic Palladium Catalyst for Enhanced Photocatalytic Water-Donating
Transfer Hydrogenation. J. Am. Chem. Soc..

[177] Zhao E., Kong W., Zoppellaro G., Yang Y., Nan B., Li L., Zhang W., Chen Z., Bakandritsos A., Wang Z. J., Beller M., Zbořil R., Chen Z. (2025). Atomic Scale Engineering of Multivalence-State
Palladium Photocatalyst for Transfer Hydrogenation with Water as a
Proton Source. Adv. Mater..

[178] Leng X., Zhang W., Lu Y., Xu L., Shen Y., Besenbacher F., Richards E., Gao H., Su R. (2026). Dual-Emulsifier Coated Photocatalyst for H_2_O_2_ Synthesis in Emulsion via Water Oxidation. Adv. Sci..

[179] Ogilby P. R. (2010). Singlet
oxygen: there is indeed something new under
the sun. Chem. Soc. Rev..

[180] Loponov K. N., Lopes J., Barlog M., Astrova E. V., Malkov A. V., Lapkin A. A. (2014). Optimization of
a Scalable Photochemical
Reactor for Reactions with Singlet Oxygen. Org.
Process Res. Dev..

[181] Huo Z., Akhsassi B., Yu J., Zheng M., Lan T., He Q., Boudon C., Xu G., Proust A., Izzet G., Ruhlmann L. (2025). Photocatalytic Recovery
of Noble Metals by Covalent
Silyl Polyoxophosphotungstate-Porphyrin Copolymers. Inorg. Chem..

[182] Xie Y., Zhang T., Guo H., Ding Z., Dong S., Chen Y., Zhang J., Guan S., Xu Z., Yu H., Bian Z. (2025). Decatungstate-Driven
Photocatalytic Pathways for Sustainable
and Cleaner Recovery of Precious Metals. Angew.
Chem., Int. Ed..

[183] Chen Y., Xu M., Wen J., Wan Y., Zhao Q., Cao X., Ding Y., Wang Z. L., Li H., Bian Z. (2021). Selective recovery of precious metals through photocatalysis. Nat. Sustainability.

[184] Zupanc A., Install J., Jereb M., Repo T. (2023). Sustainable
and Selective Modern Methods of Noble Metal Recycling. Angew. Chem., Int. Ed..

[185] Wei Y., Zhang W., Gao J. (2024). Trash or treasure?
Sustainable noble
metal recovery. Green Chem..

[192] Zhang K., Shi W., Liang W., Su R. (2025). Functionalized
Framework Materials for Light-Driven Energy and Chemical Conversions. CCS Chem..

[186] Zhang W., Yu M., Liu T., Cong M., Liu X., Yang H., Bai Y., Zhu Q., Zhang S., Gu H., Wu X., Zhang Z., Wu Y., Tian H., Li X., Zhu W.-H., Cooper A. I. (2024). Accelerated
discovery of molecular
nanojunction photocatalysts for hydrogen evolution by using automated
screening and flow synthesis. Nat. Synth..

[187] Yue H., Zhang K., Hu Y., Wang C., Huang Y., Li J., Liang W., Su R. (2025). An additive-free solid-state scalable
production of MOFs for heterogeneous catalysis. Mol. Catal..

[188] Weber S., Batey D., Cipiccia S., Stehle M., Abel K. L., Gläser R., Sheppard T. L. (2021). Hard X-Ray Nanotomography
for 3D Analysis of Coking in Nickel-Based Catalysts. Angew. Chem., Int. Ed..

[189] Vega-Peñaloza A., Mateos J., Companyó X., Escudero-Casao M., Dell’Amico L. (2021). A Rational
Approach to Organo-Photocatalysis:
Novel Designs and Structure-Property Relationships. Angew.Chem.Int. Ed..

[190] Slattery A., Wen Z., Tenblad P., Sanjosé-Orduna J., Pintossi D., den Hartog T., Noël T. (2024). Automated
self-optimization, intensification, and scale-up of photocatalysis
in flow. Science.

[191] Feng S., Wang J., Feng K., Qiao X., Zhang D., Lin H., Li Y., Rosei F., Besenbacher F., Shen Y., Pan F., Zhong J., Fan X., Su R. (2026). Sustainabl and Scalable
Flow Photochemical Conversions
Using A Labile Ligands Assembled Iron Complex. JACS Au.

